# Side-chain modulation of dithienofluorene-based copolymers to achieve high field-effect mobilities†
†Electronic supplementary information (ESI) available. See DOI: 10.1039/c6sc04129a



**DOI:** 10.1039/c6sc04129a

**Published:** 2017-02-10

**Authors:** Chia-Hao Lee, Yu-Ying Lai, Jhih-Yang Hsu, Po-Kai Huang, Yen-Ju Cheng

**Affiliations:** a Department of Applied Chemistry , National Chiao Tung University , 1001 University Road , Hsin-Chu , Taiwan . Email: yjcheng@mail.nctu.edu.tw; b Institute of Polymer Science and Engineering , National Taiwan University , Taipei , 10617 , Taiwan

## Abstract

A ladder-type dithieno[3,2-*b*:6,7-*b*′]fluorene (DTF), where the central fluorene is fused with two outer thiophene rings at its 2,3- and 6,7-junctions, is developed. The pentacyclic DTF monomers were polymerized with dithienodiketopyrrolopyrrole (DPP) acceptors to afford three alternating donor–acceptor copolymers **PDTFDPP16**, **PDTFDPP20**, and **PDTFDPP32** incorporating different aliphatic side chains (R_1_ group at DTF; R_2_ group at the DPP moieties). The side-chain variations in the polymers play a significant role in determining not only the intrinsic molecular properties but also the intermolecular packing. As evidenced by the 2-dimensional GIXS measurements, **PDTFDPP16** with octyl (R_1_) and 2-ethylhexyl (R_2_) side chains tends to align in an edge-on π-stacking orientation, whereas **PDTFDPP20** using 2-butyloctyl (R_1_) and 2-ethylhexyl (R_2_) adopts a predominately face-on orientation. **PDTFDPP32** with the bulkiest 2-butyloctyl (R_1_) and 2-octyldodecyl (R_2_) side chains shows a less ordered amorphous character. The OFET device using **PDTFDPP20** with a face-on orientation determined by GIXS measurements achieved a high hole-mobility of up to 5 cm^2^ V^–1^ s^–1^. The high rigidity and coplanarity of the DTF motifs play an important role in facilitating intramolecular 1-dimensional charge transport within the polymer backbones. The implementation of main-chain coplanarity and side-chain engineering strategies in this research provides in-depth insights into structure–property relationships for guiding development of high-mobility OFET polymers.

## Abstract 2

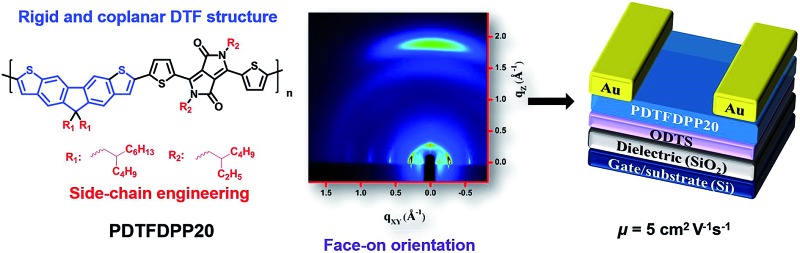
A new polymer **PDTFDPP20** based on dithieno[3,2-*b*:6,7-*b*′]fluorene (DTF) unit was developed. This polymer with a face-on orientation has achieved a high field-effect mobility up to 5 cm^2^ V^–1^ s^–1^.

## Introduction

Organic field-effect transistors (OFETs) have drawn much research attention on account of their promising advantages of good solution processibility, flexibility, and capability for large-area roll-to-roll fabrication.^1^ Solution-processable semiconducting conjugated polymers play a key role in promoting the advance of OFETs. It is generally believed that a high degree of crystallinity of the polymers with in-plane (edge-on) π-stacking is essential to achieve superior carrier transporting properties due to the existence of two charge-transporting pathways: intramolecular charge transport within the conjugated backbones, and intermolecular charge hopping through π-stacking on the condition that the polymer backbones or π-stacking directions are aligned parallel to the transistor channels.^2^ Poly(3-hexylthiophene),^3^ poly(3,3′-dialkyl-quaterthiophene),^4^ and poly(2,5-bis(thiophen-2-yl)thieno[3,2-*b*]thiophene) (pBTTT)^5^ are well-documented semiconducting polymers with high mobilities in this category. In constrast, a few emergent semiconducting donor–acceptor (D–A) copolymers lacking detectable long-range order or in-plane dominance yet exhibit mobilities close to or higher than 1 cm^2^ V^–1^ s^–1^, challenging the authority of the high crystallinity and in-plane packing model.^6^ The most representative polymer is the indacenodithiophene–benzothiadiazole (IDT–BT) copolymer exhibiting short-range and preferentially face-on crystallites, reaching a superior hole mobility of 3.6 cm^2^ V^–1^ s^–1^.^7^ McCulloch and DeLongchamp demonstrated that the backbone rigidity and coplanarity of the indacenodithiophene moiety in IDT–BT are important structural features to facilitate one-dimensional charge transport along the polymer backbones which is the primary mechanism that accounts for the exceptional OFET mobility.^7^ The high molecular weight of such polymers is also important to connect short-range crystallite domains.^8^ The understanding of structure–property relationships in the IDT–BT polymer prompts us to further implement a backbone coplanarity strategy for developing new high-mobility D–A copolymers. Ladder-type conjugated motifs with forced coplanarity therefore are suitable for this purpose.^9^ Tricyclic fluorene is a superior building block to construct a variety of organic semiconducting materials due to its good chemical stability and intrinsic hole-transporting property.^10^ Embedding a fluorene unit into a highly rigid and coplanar ladder-type conjugated framework would be a promising molecular design.^11^ To this end, we recently designed and synthesized a fluorene-based dithieno[3,2-*b*:6,7-*b*′]fluorene (DTF) in which the central fluorene is π-extended by fusing with two outer thiophene rings at its 2,3- and 6,7-junctions.^12^ Structurally analogous to IDT, the pentacyclic DTF exhibits high coplanarity and rigidity which reduces the energetic disorder of the backbone and thus enhances 1-dimensional intramolecular charge transport. Furthermore, in comparison with the IDT unit having two dialkyl sp^3^-carbon bridges in the cyclopentadiene (CP) moieties, the singly dialkyl substituted CP ring in the middle of DTF would minimize the inter- and intramolecular steric hindrance between the aliphatic side chains attached to the polymer backbones, which is beneficial to the strength of the intermolecular interactions and maintenance of main-chain coplanarity. To date, however, incorporation of DTF into D–A copolymers for organic optoelectronics has never been explored. It should also be emphasized that the choice of the aliphatic side chains attached to the polymer would dramatically influence the solubility, molecular packing and thin-film morphology which in turn would alter the mechanism of charge transport and thus the mobility.^13^ For instance, Fréchet and co-workers demonstrated that the edge-on and face-on orientations of conjugated polymers in thin films can be manipulated by the variation of their side chains.^13*d*^ In this research, newly developed electron-rich DTF units were copolymerized with electron-deficient dithienodiketopyrrolopyrrole (DPP) units to afford a new class of alternating poly(dithienofluorene-*alt*-dithienoyldiketopyrrolopyrrole) copolymers (denoted as PDTFDPPs) given that the DPP moiety has a planar conjugated bicyclic structure and strongly electron-withdrawing polar amide groups to induce strong intermolecular interactions.^14^ With an aim to control the face-on/edge-on π-stacking orientations of the polymers through side-chain engineering, three PDFTDPPs (**PDTFDPP16**, **PDTFDPP20** and **PDTFDPP32**) incorporating different aliphatic side chains were produced for systematic investigation. In spite of forming short-range crystallites with face-on π-stacking, **PDTFDPP20** with the 2-butyloctyl group on DTF and the 2-ethylhexyl group on the DPP moieties achieved an unprecedentedly high OFET mobility of 5 cm^2^ V^–1^ s^–1^.

## Results and discussion

### Design and synthesis of stannylated DTFs and PDTFDPPs

**Scheme 1:**
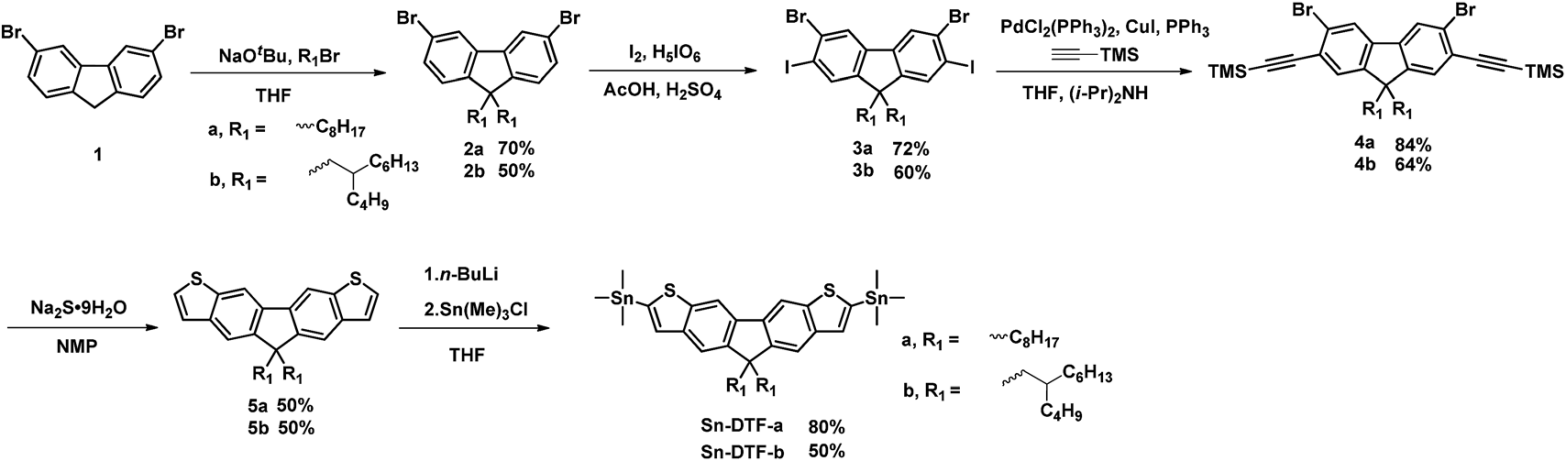
Synthesis of **Sn-DTF-a** and **Sn-DTF-b** monomers.

**Scheme 2:**
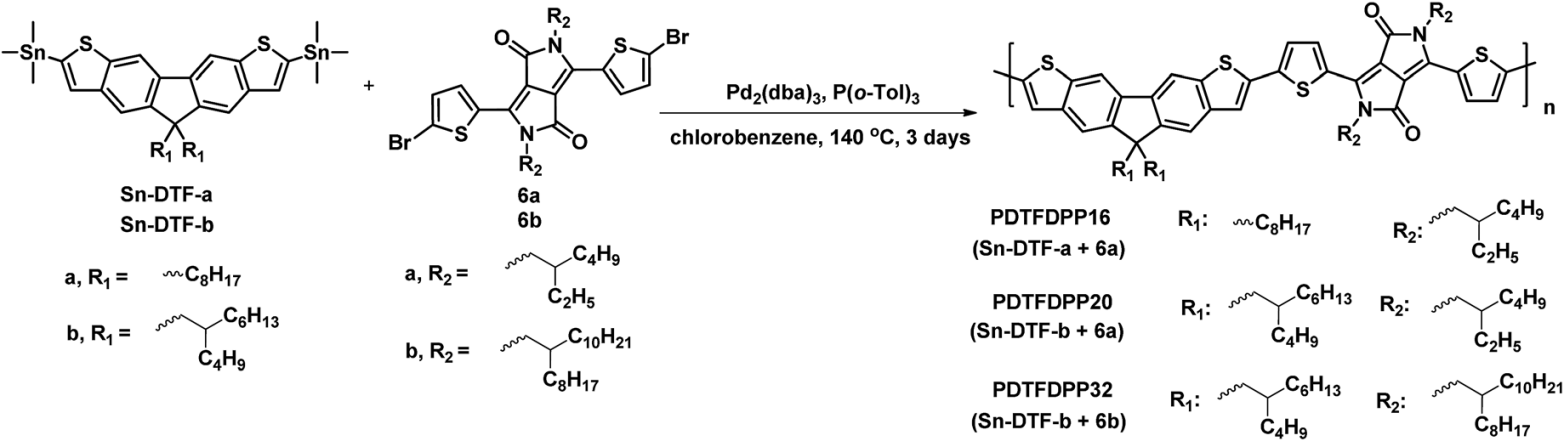
Synthesis of the three D–A copolymers **PDTFDPP16**, **PDTFDPP20**, and **PDTFDPP32**.

Linear octyl and branched 2-butyloctyl solubilizing groups are selected to be installed on the DTF unit in order to tailor the molecular packing of the resulting polymers. Synthesis of the stannylated DTF monomers is depicted in Scheme 1. 3,6-Dibromofluorene (**1**) was reacted with octyl bromide and 5-(bromomethyl)undecane under basic conditions to form 3,6-dibromo-9,9-dioctylfluorene (**2a**) and 3,6-dibromo-9,9-di(2-butyloctyl)fluorene (**2b**), respectively. Selective iodination of **2a** and **2b** at the 2,7-positions of the fluorenes yielded the key intermediates **3a** and **3b**, respectively. Sonogashira coupling of **3a** and **3b** regioselectively occurred at the 2,7-positions to afford **4a** and **4b** due to the higher reactivity of iodo over bromo. Reaction of **4a** and **4b** with Na_2_S generated the thiolate intermediates which underwent 5-*endo*-dig cyclization to form two fused thiophenes in **5a** and **5b**. Stannylation of **5a** and **5b** was carried out to afford the **Sn-DTF-a** and **Sn-DTF-b** monomers, respectively. Similarly, branched 2-ethylhexyl and 2-octyldodecyl groups with different levels of steric hindrance are introduced to the DPP monomer (**6a** and **6b**, respectively). As shown in Scheme 2, **Sn-DTF-a** and **Sn-DTF-b** were copolymerized with 2,5-di(2-ethylhexyl)-3,6-di(5-bromothiophen-2-yl)diketopyrrolopyrrole (**6a**) to furnish two alternating D–A copolymers, **PDTFDPP16** and **PDTFDPP20**, respectively (16 and 20 are the total carbon numbers of the aliphatic side chains in a repeating unit). **Sn-DTF-b** was polymerized with 2,5-di(2-octyldodecyl)-3,6-di(5-bromothiophen-2-yl)diketopyrrolopyrrole (**6b**) to yield **PDTFDPP32**. Different number average molecular weights (*M*_n_) were determined by gel permeation chromatography (GPC) to be 4.5, 68, and 32 kg mol^–1^ for **PDTFDPP16**, **PDTFDPP20**, and **PDTFDPP32**, respectively (Table 1), indicating that the magnitude of *M*_n_ is essentially governed by the aliphatic side chains flanking the DTF and DPP units. **PDTFDPP20** with branched side chains on both the DTF and DPP moieties has the highest *M*_n_, whereas **PDTFDPP16** with linear octyl groups on the DTF units shows the poorest solubility in chlorobenzene, which is responsible for its lowest *M*_n_. **PDTFDPP32** has a lower *M*_n_ than **PDTFDPP20** due to the fact that **6b** with the bulkiest 2-octyldodecyl group encounters more steric hindrance when reacting with **Sn-DFT-b** during the polymerization.

**Table 1:** Summary of the intrinsic properties of **PDTFDPP16**, **PDTFDPP20**, and **PDTFDPP32**

	*M*_n_ ^*a*^ (kDa)	PDI^*b*^	*T*_d_ ^*c*^ (°C)	*E* opt g ^*d*^ (film) (eV)	*E* ele g ^*e*^ (eV)	*λ*_max_ (nm)		*E*_HOMO_ (eV)	*E*_LUMO_ (eV)
						CHCl_3_	Film		
**PDTFDPP16**	4.5	1.2	442	1.59	1.63	655	641	–5.23	–3.60
**PDTFDPP20**	68	2.1	456	1.65	1.76	658, 707	643, 704	–5.38	–3.62
**PDTFDPP32**	32	1.8	432	1.65	1.73	643, 707	643, 704	–5.39	–3.66

^*a*^ *M*_n_ = number-average molar weight.

^*b*^PDI = polydispersity index.

^*c*^ *T*_d_ = decomposition temperature at 5% weight loss.

^*d*^ *E* opt g was estimated from the onset of UV spectrum in thin film.

^*e*^ *E* ele g was estimated from cyclic voltammetry.

### Thermal properties

**Fig. 1:**
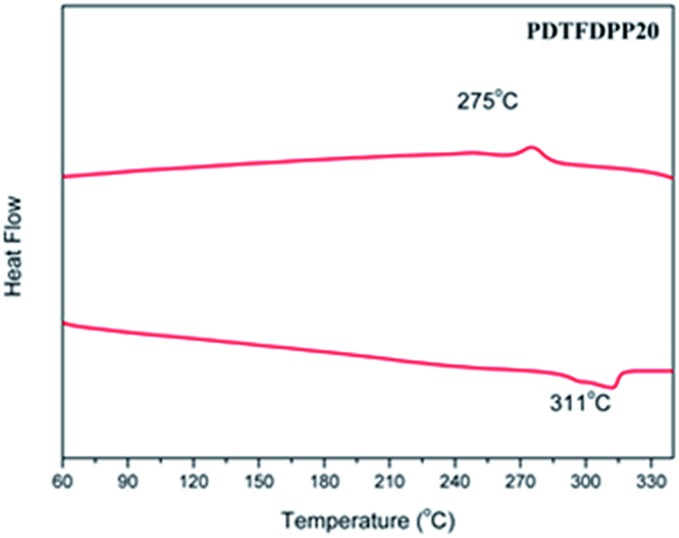
Differential scanning calorimetry of **PDTFDPP20** with a ramping rate of 10 °C min^–1^.

The thermal stability of the polymers was analyzed by thermogravimetric analysis (TGA). **PDTFDPP16**, **PDTFDPP20**, and **PDTFDPP32** exhibited sufficiently high decomposition temperatures (*T*_d_) of 442, 456, and 432 °C, respectively (Fig. S1† and Table 1). No thermal transition in the differential scanning calorimetry measurements (DSC) was observed for **PDTFDPP16** and **PDTFDPP32**, implying their less crystalline character within the range of scanning temperatures. Nevertheless, **PDTFDPP20** exhibited a melting point at 311 °C upon heating and a crystallization point at 275 °C upon cooling, implying the semicrystalline nature of **PDTFDPP20** (Fig. 1).

### Optical properties

**Fig. 2:**
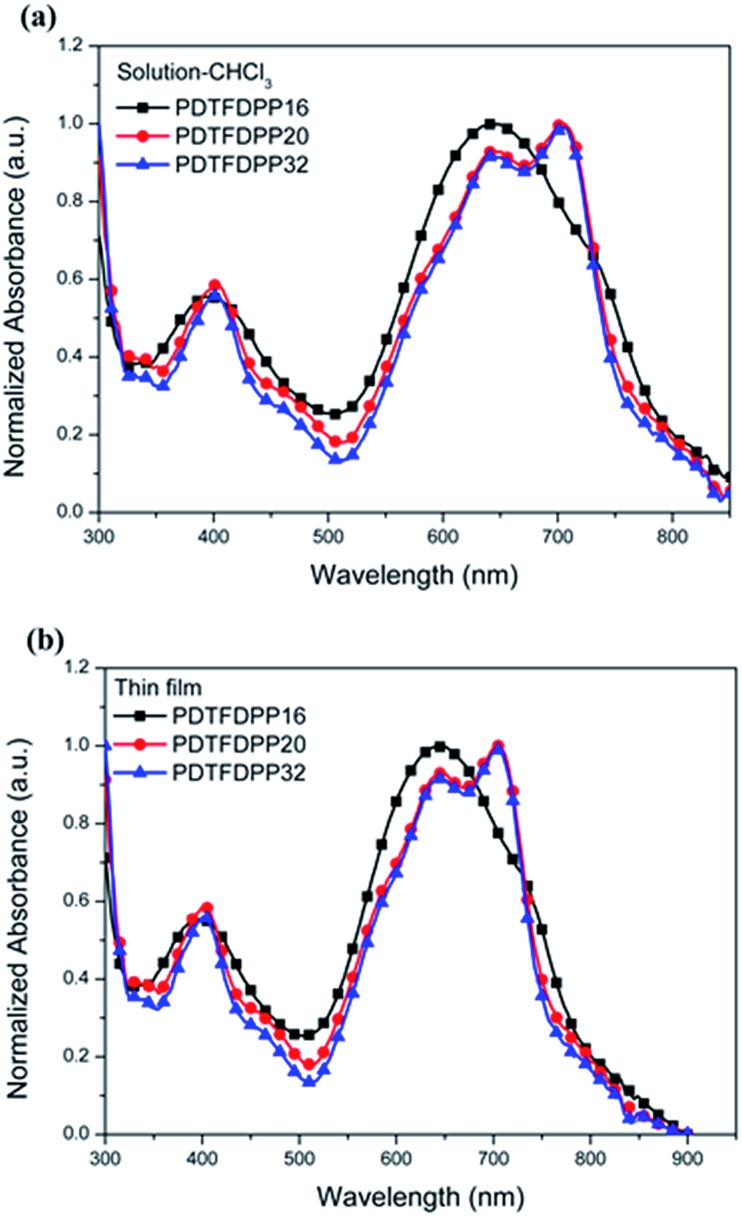
Normalized UV-vis absorption spectra of **PDTFDPP16**, **PDTFDPP20**, and **PDTFDPP32** in (a) chloroform solutions; (b) thin films.

The UV-vis absorption spectra in CHCl_3_ and thin film are depicted in Fig. 2 and summarized in Table 1. It is expected that the three polymers should possess analogous absorption profiles due to their identical backbone composition. Nevertheless, **PDTFDPP20** and **PDTFDPP32** exhibited a pronounced vibronic structure, which is much less noticeable for **PDTFDPP16**. **PDTFDPP20** and **PDTFDPP32** showed lower-lying HOMO energy levels of –5.38 and –5.39 eV, respectively, while **PDTFDPP16** exhibited a higher HOMO level of –5.23 eV. The higher HOMO level may be associated with the linear side chain in **PDTFDPP16**, which results in less distorted polymer chains. **PDTFDPP32** might already reach a saturation of the effective conjugated length. Therefore, **PDTFDPP20** with higher molecular weight shows an almost identical absorption profile to **PDTFDPP32**.

### Electrochemical properties

**Fig. 3:**
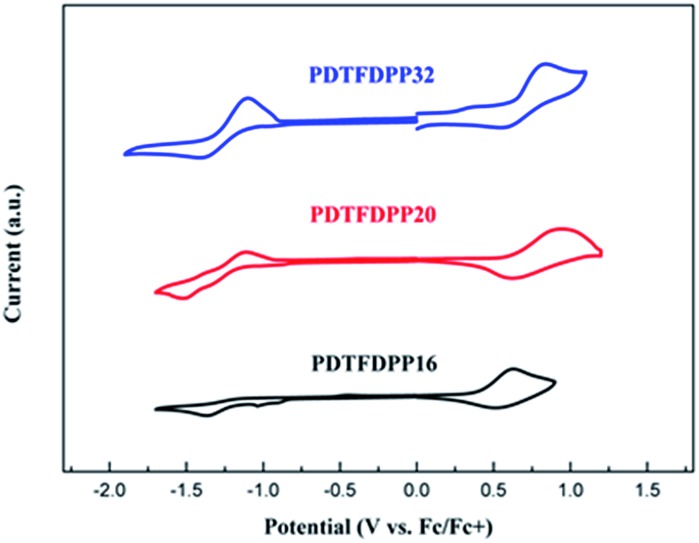
Cyclic voltammograms of **PDTFDPP16**, **PDTFDPP20**, and **PDTFDPP32** in thin films at a scan rate of 100 mV s^–1^.

Cyclic voltammetry (CV) was employed to determine the highest occupied molecular orbital (HOMO) and lowest unoccupied molecular orbital (LUMO) energies of the polymers (Fig. 3, Table 1). **PDTFDPP20** and **PDTFDPP32** showed lower-lying HOMO energy levels of –5.38 and –5.39 eV, respectively, while **PDTFDPP16** exhibited a higher HOMO level of –5.23 eV. This discrepancy might result from the variation in the HOMO electron-density distribution. As for the LUMO, the three polymers have comparable values, which approximate to –3.60 eV.

### GIXS measurements

**Fig. 4:**
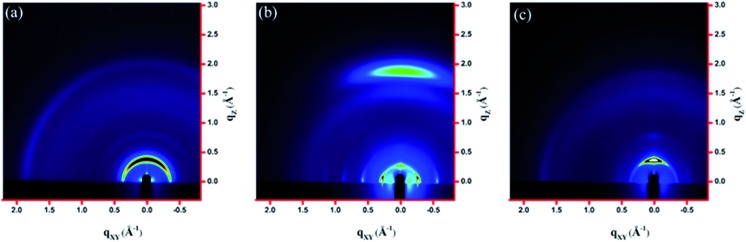
2-Dimensional GIXS images of the thin films (a) **PDTFDPP16**, (b) **PDTFDPP20**, and (c) **PDTFDPP32** on the ODTS-treated Si/SiO_2_ substrate.

**Fig. 5:**
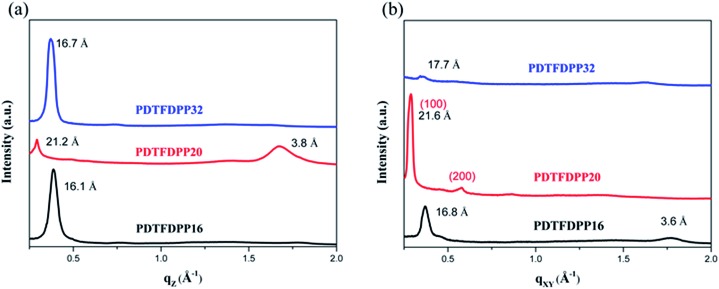
1-Dimensional (a) out-of-plane and (b) in-plane GIXS patterns of **PDTFDPP16**, **PDTFDPP20**, and **PDTFDPP32**. The spacings are listed near the peaks.

The molecular packing of the thin films on an Si/SiO_2_ substrate modified by a self-assembled monolayer (SAM) of octadecyltrichlorosilane (ODTS) was investigated using 2-dimensional grazing incidence X-ray scattering (GIXS). The 2-D GIXS images and the corresponding 1-dimensional out-of-plane (*q*_*z*_) and in-plane (*q*_*xy*_) diffraction patterns of the polymer thin films annealed at 200 °C for 10 minutes are illustrated in Fig. 4 and 5, respectively. The **PDTFDPP20** film exhibited in-plane (*h*00) signals assigned to a lamellar spacing (*d*_l_) of *ca.* 21.6 Å corresponding to side-chain interdigitation (Fig. 5b). Meanwhile, **PDTFDPP20** also showed an obvious (010) out-of-plane peak at *q*_*z*_ = 1.7 Å^–1^ corresponding to periodic π–π stacking with a distance (*d*_π_) of *ca.* 3.8 Å between the two facing conjugated backbones (Fig. 5a). The out-of-plane (010) peak also reveals that the **PDTFDPP20** adopts a predominately face-on orientation with the backbone plane approximately parallel to the substrate. Conversely, judging from the out-of-plane lamellar stacking (*q*_*z*_ = 0.4 Å^–1^ corresponding to *d*_l_ = 16.1 Å, Fig. 5a) and the in-plane π-stacking (*q*_*xy*_ = 1.7 Å^–1^ corresponding to *d*_π_ = 3.6 Å, Fig. 5b) peaks, **PDTFDPP16** tends to align preferably in an edge-on π-stacking orientation with the polymer backbone roughly perpendicular to the substrate.

As for **PDTFDPP32**, no evident π-stacking peak could be found in the direction of either *q*_*z*_ or *q*_*xy*_, indicative of its amorphous character.

**Fig. 6:**
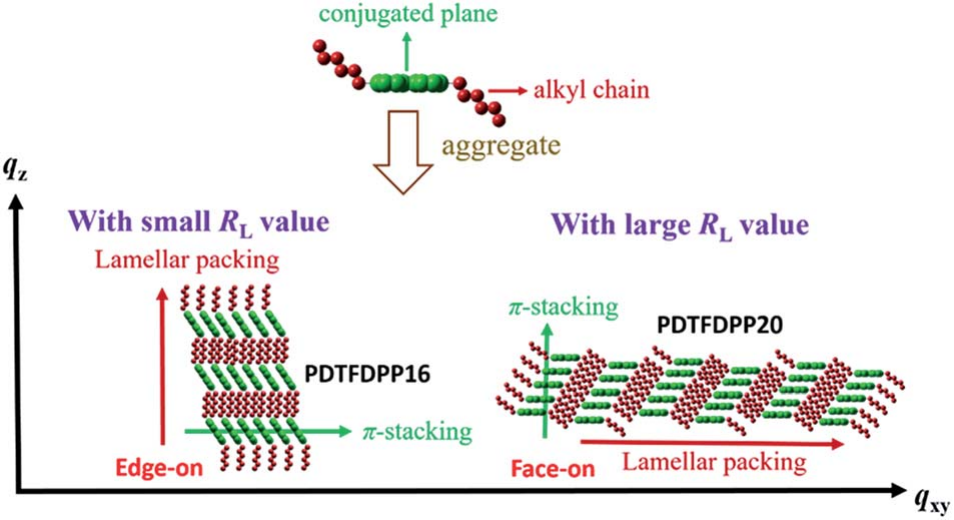
Illustration of the 2-dimensional lamellar and π-stacking crystallites of **PDTFDPP16** and **PDTFDPP20** with *R*_L_ of 2.5 and 5.7, respectively.

The correlation length (*L*_c_) derived from the full width at half maximum (FWHM) of a X-ray diffraction peak in GIXS based on Scherrer's equation can be used to estimate the size of a crystallite.^1f,7,13^ ^*d*^ By combining the lamellar-stacking diffraction peak in the *q*_*xy*_ or *q*_*z*_ direction and the π-stacking peak in the corresponding perpendicular direction (*i.e. q*_*z*_ or *q*_*xy*_, respectively) in the 2D-GIXS images, we can approximately acquire the vertical and horizontal correlation lengths (*L*_c_) of a 2-dimensional crystallite. We realized that the ratio (*R*_L_) of lamellar stacking *L*_c_ to π-stacking *L*_c_ is an important parameter in dictating the face-on/edge-on orientations of the PDTFDPPs. The **PDTFDPP16** film with a π-stacking *L*_c_ of 4.0 nm (*q*_*xy*_) and a lamellar *L*_c_ of 10.1 nm (*q*_*z*_) leads to a *R*_L_ of 2.5, while the **PDTFDPP20** film with a π-stacking *L*_c_ of 3.4 nm (*q*_*z*_) and a lamellar *L*_c_ of 19.5 nm (*q*_*xy*_) yields a *R*_L_ of 5.7. The relevant parameters are summarized in Table 2. It can be reasonably inferred that a crystallite with a smaller *R*_L_ value prefers to align in an edge-on π-stacking orientation perpendicular to the substrate. Increasing the *R*_L_ value by lengthening the lamellar-stacking dimension changes the π-stacking from an edge-on alignment to a face-on orientation. The 2-dimensional molecular packing of **PDTFDPP16** and **PDTFDPP20** is illustrated in Fig. 6. This argument is also valid for other well-investigated polymer systems. It should also be noted that other factors such as side chain density,^15^ solvent selection^16^ and the branching point position of the side chain^17^ might also play important roles in determining the crystalline orientation. For example, P3HT and PBTTT-C12 (ref. 5*a*) with smaller *R*_L_ values of 2.03 and 1.90, respectively, show edge-on orientations, whereas PDPP3F-BO with a large *R*_L_ value of 7.86 favors the face-on packing.^13*d*^ This observation is also consistent with the fact that a nanorod with a high aspect ratio (length divided by width) fails to stand vertically on a substrate.^18^ Additional GIXS data evidencing this argument are provided in the ESI.†

**Table 2:** Stacking parameters of the **PDTFDPP16** and **PDTFDPP20** thin films

	π-Stacking		Lamellar stacking		*R*_L_ ^*c*^
	*d* ^*a*^ (Å)	*L*_c_ ^*b*^ (nm)	*d* ^*a*^ (Å)	*L*_c_ ^*b*^ (nm)	
**PDTFDPP16**	3.6	4.0	16.8	10.1	2.5
**PDTFDPP20**	3.8	3.4	21.6	19.5	5.7

^*a*^Spacing of diffraction peak.

^*b*^Correlation length based on Scherrer's equation.

^*c*^Ratio of *L*_c_ (lamellar spacing) to *L*_c_ (π-stacking).

**Fig. 7:**
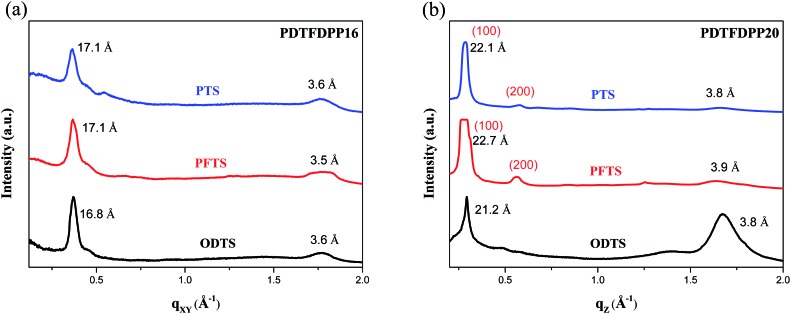
1-Dimensional GIXS patterns of (a) **PDTFDPP16** in the *q*_*xy*_ and (b) **PDTFDPP20** in the *q*_*z*_ direction on the SAM layer of PTS, PFTS and ODTS.

In addition to the crystallite dimensions, the interactions between the polymers and underlying self-assembled monolayer (SAM) on the Si/SiO_2_ substrate may also influence the polymer orientation. Thin films on trichloro(phenethyl)silane (PTS) and trichloro(1*H*,1*H*,2*H*,2*H*-perfluorooctyl)silane (PFTS) SAM layers were thus prepared and examined in comparison with ODTS. The GIXS measurements of **PDTFDPP16** in the *q*_*xy*_ direction (Fig. 7) reveal that the edge-on π-stacking remains essentially intact regardless of the SAM layer; however, the original face-on orientation of **PDTFDPP20** on ODTS in the *q*_*z*_ direction is almost diminished when the SAM layer is changed to PTS or PFTS.

The results indicate that the formation of face-on π-stacking is highly dependent on the underlying SAM layer, which may be attributed to the more intimate contact between the face-on π-conjugated plane and the underlying substrate. It should be also mentioned that the GIXS measurements only reflect the bulk orientations of the thin films. The polymer might adopt a different orientation at the semiconductor/dielectric interface which is the channel area where the charge transport occurs.^19^

### Theoretical calculations

**Fig. 8:**
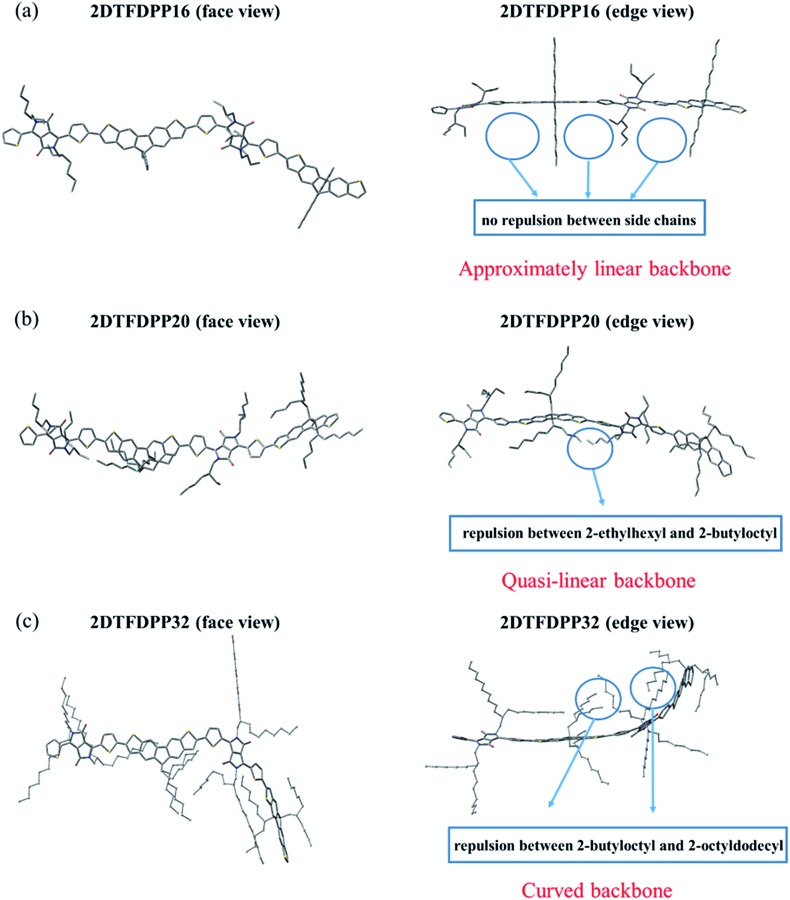
Optimized geometry of dimeric structures (a) 2DTFDPP16; (b) 2DTFDPP20; (c) 2DTFDPP32 (face view and edge view).

In order to gain insight into the steric effect derived from different side chains installed on PDTFDPP, our own n-layered integrated molecular orbital and molecular mechanics (ONIOM) calculations were performed. ONIOM approaches were developed to compute large molecules with high accuracy and reduced computational time.^20^ Within the target structure, two or three layers are defined and treated at different levels of theory. In this study, dimeric structures, 2DTFDPP16, 2DTFDPP20, and 2DTFDPP32 were used as the model compounds for simulating **PDTFDPP16**, **PDTFDPP20**, and **PDTFDPP32**, respectively. Geometry optimization was carried out with the B3LYP/6-31G(d) level of theory applied to the conjugated systems and the universal force field (UFF) applied to the alkyl side chains. The optimized structures are depicted in Fig. 8. It is evident from the edge-viewed structures that 2DTFDPP16 has the most planar and linear conjugated backbone among the three model compounds due to the low steric repulsion between the octyl and 2-ethylhexyl side chains in 2DTFDPP16. It is envisioned that **PDTFDPP16** would have strong intermolecular π–π stacking, leading to poor solubility during polymerization and thus would have the lowest *M*_n_. In contrast, steric repulsion between the 2-butyloctyl and 2-octyldodecyl groups in 2DFTDPP32 distorts its main-chain coplanarity and linearity, which is disadvantageous for intermolecular π–π stacking (Fig. 8c). The monomeric unit of **PDTFDPP32** has the highest molecular weight among all; however, the *M*_n_ of **PDTFDPP32** is significantly lower than that of **PDTFDPP20**. This side-chain repulsion obstructs the cross-coupling reaction between two monomeric units, accounting for the modest *M*_n_ of **PDTFDPP32**. 2DTFDPP20 shows a quasi-linear backbone. The 2-butyloctyl and 2-ethylhexyl moieties in **PDTFDPP20** are sufficient to solubilize the resulting polymer without hampering the polymerization, leading to the highest *M*_n_. Furthermore, the curved main-chain structure of 2DTFDPP32 is associated with the amorphous character of **PDTFDPP32** suggested by the 2D-GIXS. As mentioned previously, the planar and linear conjugated plane might allow **PDTFDPP16** to form a longer π-stacking *L*_c_ than **PDTFDPP20**. On the other hand, it is envisaged that **PDTFDPP20** with the dense aliphatic chains would assemble into a longer lamellar packing *L*_c_ than **PDTFDPP16**. Accordingly, the variation in the crystallite dimensions results in the disparity in the thin-film orientation between **PDTFDPP16** and **PDTFDPP20**.

### Transistor characterization

**Fig. 9:**
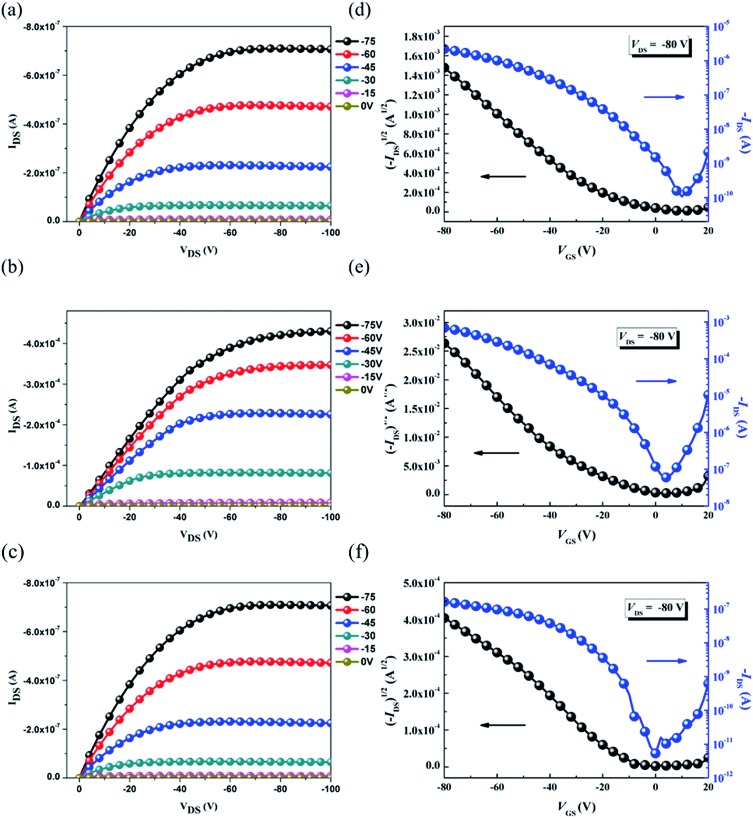
Typical output curves (a, b and c) and transfer plots (d, e and f) of the bottom-gate/top-contact OFET devices for **PDTFDPP16**, **PDTFDPP20**, and **PDTFDPP32**, respectively.

The OFET mobilities of the DTF-based polymers were measured by devices using a bottom-gate/top-contact configuration with evaporated gold source/drain electrodes (40 nm in thickness). The OFET devices used SiO_2_ as the gate dielectric whose surfaces were modified with a SAM layer of ODTS. All of the output and transfer plots of the devices exhibited typical p-type OFET characteristics (Fig. 9). The hole mobilities are calculated from the transfer characteristics of the devices in the saturation regime. As summarized in Table 3, the hole mobility of the **PDTFDPP16** device was 1.20 × 10^–2^ cm^2^ V^–1^ s^–1^ with an on–off ratio of 4.98 × 10^5^. **PDTFDPP32** with more carbons in the aliphatic side chains exhibited the lowest mobility of 1.04 × 10^–3^ cm^2^ V^–1^ s^–1^. **PDTFDPP20** achieved the highest hole mobility reaching 5.00 cm^2^ V^–1^ s^–1^ in the saturated regime. The mobility extracted from the linear regime showed an even higher value of 5.26 cm^2^ V^–1^ s^–1^. GIXS measurements reveal that **PDTFDPP20** formed short-range and face-on crystallites in the thin film which is in opposition to the conventional point of view that long-range crystallinity and edge-on π-stacking are essential for high-performance OFET materials. According to the work carried out by Salleo and co-workers, efficient charge transport between the short-range crystallites in the polymer thin film can be realized on the condition that they are inter-connected.^8^ Compared to **PDTFDPP16**, the much higher *M*_n_ (68 kg mol^–1^) of **PDTFDPP20** not only enhances intrachain charge transport but also enables sufficient connection between the different crystalline domains to realize a high OFET mobility. More importantly, the rigidity and coplanarity of the DTF units facilitate intramolecular 1-dimensional charge transport within the polymer backbones. Charge transport to adjacent chains *via* π-stack hopping is supposed to be the minor pathway.

**Table 3:** Bottom-gate/top-contact OFET characteristics of the polymer thin films

Polymer	SAM layer	Annealing temp (°C)	Mobility^*a*^ (cm^2^ V^–1^ s^–1^)	On/off ratio	*V*_t_ (V)
**PDTFDPP16**	ODTS	200	1.20 × 10^–2^	4.98 × 10^5^	–19.0
**PDTFDPP20**	ODTS	240	5.00	8.67 × 10^5^	–26.0
**PDTFDPP20**	ODTS	200	5.26^*b*^	1.57 × 10^6^	–1.6
**PDTFDPP32**	ODTS	Pristine	1.04 × 10^–3^	3.85 × 10^4^	–13.1

^*a*^Saturated mobility.

^*b*^Linear mobility with *V*_ds_ of –20 V.

**Fig. 10:**
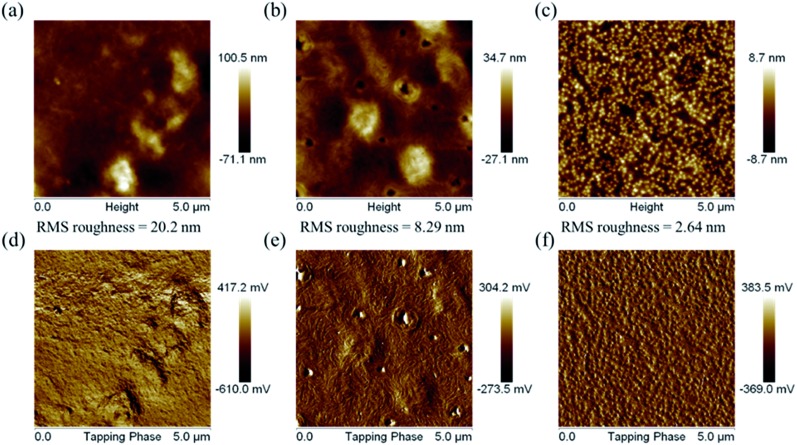
Atomic force microscopy height (a, b and c) and phase (d, e and f) images for **PDTFDPP16**, **PDTFDPP20**, and **PDTFDPP32**, respectively.

The surface morphologies of the PDTFDPPs were investigated by atomic force microscopy (AFM). As shown in Fig. 10, **PDTFDPP16** exhibited obvious aggregation with a high RMS roughness of 20.2 nm due to its poor solubility in chloroform. However, **PDTFDPP32** with better solubility showed a more homogeneous thin-film structure with a dramatically reduced roughness of 2.64 nm. In particular, **PDTFDPP20** with a roughness of 8.29 nm exhibited a fibrous texture in its phase image, which might be also associated with its highest charge mobility. These results again demonstrate that side-chain engineering of PDTFDPPs plays a pivotal role in determining the thin-film morphology.

## Conclusions

We designed and synthesized a pentacyclic dithieno[3,2-*b*:6,7-*b*′]fluorene (DTF) where the central fluorene is π-extended by fusing with two outer thiophene rings at the 2,3- and 6,7-junctions. The newly developed DTF units were copolymerized with the DPP acceptors to yield three D–A copolymers **PDTFDPP16**, **PDTFDPP20**, **PDTFDPP32** with different aliphatic side chains. The side-chain variations in the polymers play a key role in determining not only the intrinsic molecular properties but also the intermolecular packing. The face-on/edge-on π-stacking of the PDTFDPP-based polymers are associated with the ratio (*R*_L_) of the lamellar stacking correlation length to the π-stacking correlation length of the crystallites. Based on GIXS thin film measurements, **PDTFDPP16** with a smaller *R*_L_ tends to align in an edge-on π-stacking orientation, whereas **PDTFDPP20** with a large *R*_L_ adopts a predominately face-on orientation. Bottom-gate/top-contact OFET devices using **PDTFDPP20** achieved high hole mobilities of up to 5 cm^2^ V^–1^ s^–1^. To the best of our knowledge, this value represents the highest p-type mobility for solution-processable conjugated polymers with a face-on orientation determined by GIXS. The rigidity and coplanarity of the DTF framework facilitate intramolecular 1-dimensional charge transport within the polymer backbones. A sufficiently high molecular weight to connect the short-range crystalline domains in **PDTFDPP20** is also crucial for efficient charge transport.

This research verifies that edge-on dominance and long-range order to achieve high mobility might not be obligatory as long as the short-range crystallites can be interconnected and efficient intrachain 1D-charge transport along the backbone can be established. This work, implementing main-chain coplanarity and side-chain variations, will provide a new direction to guide the development of new high-mobility semiconducting materials.
