# Supplementary material for: Side-chain modulation of dithienofluorene-based copolymers to achieve high field-effect mobilities
Source: Chem Sci. 2017 Feb 10;8(4):2942–51. doi: 10.1039/c6sc04129a (PMC5376713; doi:10.1039/c6sc04129a)
Supplement: Supplementary file 1 [file SC-008-C6SC04129A-s001.pdf]

Electronic Supplementary Material (ESI) for Chemical Science.
This journal is © The Royal Society of Chemistry 2017

Supporting Information

Side-Chain Modulation of Dithienofluorene-Based Copolymers to Achieve High Field-Effect Mobility

Chia-Hao Lee,[a] Yu-Ying Lai,[b] Jhih-Yang Hsu,[a] Po-Kai Huang,[a] and Yen-Ju Cheng*[a]

[a]Department of Applied Chemistry, National Chiao Tung University, 1001 University Road, Hsin-Chu, Taiwan

[b]Institute of Polymer Science and Engineering, National Taiwan University, Taipei, 10617, Taiwan

Table of Contents

1. General Measurement and Characterization. S1
2. OFET Fabrication. S2
3. Experimental Procedures. S3
4. Thermogravimertic Analysis (TGA) S8
5. Grazing Incidence X-ray Scattering (GIXS) S9
6. OFET Data……………………………………………………………..S10
7. Polymer Information……………………………………………………...S11
8. Atomic Force Microscopy (AFM) ……………………………………S11
9. ^{1}H and ^{13}C NMR Spectra S12
10. Computational Details. S27
11. References...S59

1. General Measurement and Characterization.

^{1}H and ^{13}C NMR spectra were measured using Varian 400 MHz instrument spectrometer and obtained in deuterated chloroform ($CDCl_3$) with TMS as internal reference unless otherwise stated, and chemical shifts (δ) are reported in parts per million. Molecular weights of the polymers were determined by GPC with a PN5300 of Postnova using THF as a solvent and calibrated with polystyrene standards. Absorption spectra were taken on a HP8453 UV-vis spectrophotometer. Differential scanning calorimetery (DSC) was conducted on a TA Q200 Instrument under nitrogen atmosphere at a heating/cooling rate of 10 °C/min. Thermogravimetric analysis (TGA) was recorded on a Perkin-Elmer Pyris under nitrogen atmosphere at a heating rate of 10 °C/min. Electrochemical cyclic voltammetry was conducted on a CH instruments electrochemical analyzer. A carbon glass was used as the working electrode and a Ag/AgCl electrode as the reference electrode, while 0.1 M tetrabutylammonium hexafluorophosphate in acetonitrile was the electrolyte. CV curves were calibrated using ferrocence as the standard, whose HOMO is set at −4.8 eV with respect to zero vacuum level. The HOMO energy levels were obtained from the equation HOMO = $-(E_{ox}^{onset} - E_{(ferrocene)}^{onset} + 4.8)$ eV. The LUMO levels were obtained from the equation LUMO = $-(E_{red}^{onset} - E_{(ferrocene)}^{onset} + 4.8)$ eV. GIXS experiments were conducted at National Synchrotron Radiation Research Center (NSRRC) on beamline BL23A in Taiwan. The samples were irradiated with an X-ray energy of 10.09 keV (λ = 1.23 Å) at a fixed incident angle of 0.08° through a coupled double crystal Si(111)/multilayer (Mo/B4C) monochromator, and the GIXS patterns were recorded on a 2D image detector (Pilatus 1M-F area detector). The polymer films for GIXS measurement were prepared under identical conditions used for the OFET devices. Atomic Force Microscopy for surface topography was investigated by Veeco Nanoscope 3100 and standard tips (type Tap 300; L, 135 m; FREQ, 300 MHz; k, 40 N/m).

2. OFET Fabrication.

325 nm thick SiO_2 was deposited on the n-doped silicon wafer (Ci = 11 nF cm^{-2}). The substrates were rinsed by sulfuric acid and hydrogen peroxide (30% solution in water) (3:1, volume ratio) at room temperature for 1 h, followed by 15 min of sonication in pure water. The substrates were heated on a hot plate at 150 °C to remove water in a glovebox, followed by UV-ozone treatment for 30 min. The SiO_2 was immersed in a octadecyltrichlorosilane (ODTS):toluene solution (1:100, volume ratio) for 3 h. The surface of the ODTS-treated SiO_2/Si substrates was washed by acetone and heated for 1 h at 100°C. Thin films (40−60 nm in thickness) of polymers were deposited on ODTS-treated SiO_2/Si substrates by spin-coating (1000 rpm) their hot $CHCl_3$ solutions (10 mg/mL). The treatment conditions of trichloro(phenethyl)silane (PTS) and trichloro(1H,1H,2H,2H-perfluorooctyl)silane (PFTS) are identical with that of ODTS. Thermal annealing was then conducted at 200 °C for 10 min. Gold source and drain contacts (40 nm in thickness) were deposited by vacuum evaporation on the polymer layer to complete the bottom-gate/top-contact OFET devices. Electrical measurements of all OFET devices were carried out at room temperature in air on a 4156C (Agilent Technologies). The field-effect mobility was calculated in the saturation and linear regime by using the equation $I_{ds} = (\mu W C_i/2L)(V_g - V_t)^2$ and $I_{ds}=(W/L)\mu C_i V_{ds}\ (V_g\text{-}V_t\text{-}1/2\ V_{ds})$, respectively, where I_{ds} is the drain-source current, μ is the field-effect mobility, W is the channel width (1 mm), L is the channel length (100 μm), C_i is the capacitance per unit area of the gate dielectric layer, V_g is the gate voltage, and V_t is threshold voltage.

3. Experimental Procedures.

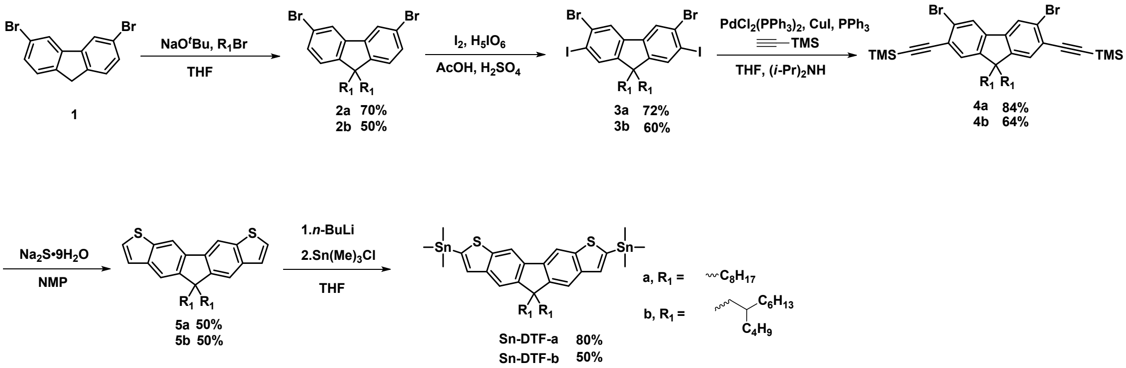

Scheme S1. Synthesis of **Sn-DTF-a** and **Sn-DTF-b** monomers.

Compound **1** and **2a-5a** were synthesized as reported.[S1]

Synthesis of 2b. To a mixture of compound **1** (2 g, 6.17 mmol) and 1-bromo-2-butyloctane (3.8 g, 15.37 mmol) was added slowly a THF (100 ml) solution of sodium *tert*-butoxide (2.37 g, 24.66 mmol) at ice bath under nitrogen atmosphere. The reaction mixture was stirred for 16 hours at room temperature, evaporated under vacuum, and extracted with ethyl acetate (250 mL × 3) and water (250 mL). The combined organic layer was dried over $MgSO_4$. After filtration and removal of the solvent, the excess amount of 1-bromo-2-butyloctane was removed by distillation under reduced pressure. The residue was purified by column chromatography on silica gel (hexane) to give a transparent oil **2b**. (2.05 g, 50%): ^{1}H NMR ($CDCl_3$, 400 MHz): δ 7.77 (d, J = 1.6 Hz, 2 H), 7.40 (dd, J = 8 Hz, J = 1.6 Hz, 2 H), 7.23 (d, J = 8 Hz, 2 H), 1.93 (d, J = 5.2 Hz, 4 H), 1.21-1.16 (m, 4 H), 1.06-1.02 (m, 4 H), 0.94–0.81 (m, 24 H), 0.71 (m, 12 H), 0.46 (m, 2 H); ^{13}C NMR ($CDCl_3$, 100 MHz): δ 149.5, 142.1, 129.9, 125.6, 123.1, 120.8, 54.8, 44.8, 34.6, 34.4, 33.2, 31.7, 29.3, 28.1, 25.8, 22.7, 22.6, 14.1, 14.0; MS (EI, $C_{37}H_{56}Br_2^{+\cdot}$): calcd, 658.2743; found, 658.2766.

Synthesis of 3b. To a 100 ml two-neck round-bottom flask connected to a balloon filled with nitrogen were added compound **2b** (1.20 g, 1.82 mmol), iodine (0.81 g, 3.20

mmol), periodic acid (0.21 g, 0.92 mmol), 1,2-dichloroethane (10 ml), acetic acid (90 ml) and fuming sulfuric acid (2 ml). The resulting mixture was heated at 80 ℃ for 4 hours, cooled to room temperature, and extracted with ethyl acetate (100 mL × 3) and water (100 mL). The combined organic layer was dried over $MgSO_4$, filtrated, and concentrated *in vacuo*. The residue was purified by column chromatography on silica gel (hexane) to give a yellow oil **3b** (1.0 g, 60%): ^{1}H NMR ($CDCl_3$, 400 MHz): δ 7.91 (s, 2 H), 7.86 (s, 2 H), 1.90 (d, J = 5.2 Hz, 4 H), 1.26-1.20 (m, 4 H), 1.18-1.08 (m, 4 H), 1.00-0.82 (m, 24 H), 0.77 (m, 12 H), 0.46 (m, 2 H); ^{13}C NMR ($CDCl_3$, 100 MHz): δ 150.8, 141.1, 136.0, 128.1, 123.9, 99.7, 54.9, 44.3, 34.9, 34.7, 33.4, 31.8, 29.4, 28.3, 26.0, 22.8, 22.8, 14.2, 14.2; HRMS (field desorption (FD), $C_{37}H_{54}Br_2I_2^{+\cdot}$): calcd, 910.0676; found, 910.0678.

Synthesis of 4b. To a degassed toluene (10 mL) solution of compound **3b** (1 g, 1.10 mmol) and diisoproprylamine (6 mL), ethynyltrimethylsilane (0.33 mL, 2.32 mmol), $PdCl_2(PPh_3)_2$ (15.0 mg, 0.02 mmol), and CuI (8.5 mg, 0.04 mmol) were added. The resulting mixture was stirred for 1 hour at room temperature, diluted with water (50 mL), extracted with ethyl acetate (50 mL × 3), dried over $MgSO_4$, and concentrated under vacuum. The residue was purified by column chromatography on silica gel (hexane) to give a transparent oil **4b** (600 mg, 64%): ^{1}H NMR ($CDCl_3$, 400 MHz): δ 7.83 (s, 2 H), 7.46 (s, 2 H), 1.92 (d, J = 5.6 Hz, 4 H), 1.23-1.18 (m, 4 H), 1.09-1.07 (m, 4 H), 0.95–0.83 (m, 24 H), 0.73 (m, 12 H), 0.50 (br, 2 H), 0.28 (s, 18 H); ^{13}C NMR ($CDCl_3$, 100 MHz): δ 149.8, 141.0, 128.9, 124.6, 124.0, 123.8, 103.7, 100.0, 54.8, 44.5, 34.6, 34.4, 33.3, 31.7, 29.4, 28.2, 28.2, 25.8, 22.8, 22.7, 14.1, 14.0, -0.2; HRMS (FD, $C_{47}H_{72}Br_2Si_2^{+\cdot}$): calcd, 850.3533; found, 850.3524.

Synthesis of 5b. To a suspension of sodium sulfide nonahydrate (780 mg, 3.25 mmol) in NMP (30 mL) was added compound **4b** (600 mg, 0.70 mmol). The mixture was then heated at 195 °C for 12 hours, poured into saturated aqueous ammonium chloride

solution (100 mL), extracted with ethyl acetate (100 mL × 3), dried over $MgSO_4$, and concentrated under vacuum. The residue was purified by column chromatography on silica gel (hexane/ethyl acetate, v/v, 80/1) to give a light yellow solid **5b** (215 mg, 50%): ^{1}H NMR ($CDCl_3$, 400 MHz): δ 8.24 (s, 2 H), 7.81 (s, 2 H), 7.42 (d, J = 5.2 Hz, 2 H), 7.35 (d, J = 5.2 Hz, 2 H), 2.12 (d, J = 4 Hz, 4 H), 1.09-1.03 (m, 4 H), 0.97–0.70 (m, 36 H), 0.61 (t, J = 6.6 Hz, 6 H); ^{13}C NMR ($CDCl_3$, 100 MHz): δ 147.9, 139.0, 138.5, 125.9, 123.8, 119.0, 119.0, 113.1, 53.8, 46.2, 34.6, 34.5, 34.4, 33.2, 31.7, 29.3, 28.1, 28.1, 25.8, 25.7, 22.7, 22.6, 14.1, 13.9; HRMS (EI, $C_{41}H_{58}S_2^{+\cdot}$): calcd, 614.3974; found, 614.3990.

Synthesis of Sn-DTF-a. To an anhydrous THF (30 mL) solution of **5a** (450 mg, 0.90 mmol) was added *n*-butyllithium (1 ml, 2.5 M) slowly at –78 °C. The stirring was continued for 40 min at –78 °C and trimethyltin chloride (2.7 ml, 1 M) was added. The mixture was warmed to room temperature gradually and stirred for 12 h. It was quenched with saturated NH_4Cl solution (10 mL), cooled to room temperature, and extracted with ethyl acetate (200 mL × 3) and water (200 mL). The combined organic layer was dried over $MgSO_4$, filtrated, and concentrated *in vacuo*. A transparent oil **Sn-DTF-a** was obtained (600 mg, 80%). ^{1}H NMR ($CDCl_3$, 400 MHz): δ 8.21 (s, 2 H), 7.70 (s, 2 H), 7.44 (s, 2 H), 2.03 (br, 4 H), 1.17–1.00 (m, 20 H), 0.78 (t, J = 7.2 Hz, 6 H), 0.59 (br, 4 H), 0.44 (s, 18 H); ^{13}C NMR ($CDCl_3$, 100 MHz): δ 148.3, 143.6, 140.8, 140.3, 138.0, 132.0, 116.6, 112.6, 53.8, 41.9, 31.8, 30.1, 29.3, 29.2, 23.8, 22.6, 14.0, –8.4; HRMS (FD, $C_{39}H_{58}S_2Sn_2^{+\cdot}$): calcd, 830.2018; found, 830.2014.

Synthesis of Sn-DTF-b. To an anhydrous THF (20 mL) solution of **5b** (260 mg, 0.42 mmol) was added *n*-butyllithium (0.43 mL, 2.5 M) slowly at –78 °C. The stirring was continued for 40 min at 78 °C and trimethyltin chloride (1.27 ml, 1 M) was added. The mixture was warmed to room temperature gradually and stirred for 12 h. It was quenched with saturated NH_4Cl solution (10 mL), cooled to room temperature, and

extracted with ethyl acetate (200 mL × 3) and water (200 mL). The combined organic layer was dried over $MgSO_4$, filtrated, and concentrated *in vacuo*. A transparent oil **Sn-DTF-b** was obtained (200 mg, 50%). ^1H NMR ($CDCl_3$, 400 MHz): δ 8.22 (s, 2 H), 7.74 (s, 2 H), 7.41 (s, 2 H), 2.08 (d, J = 3.2 Hz, 4 H), 1.04-1.01 (m, 4 H) 1.04–0.58 (m, 42 H), 0.45 (s, 18 H); ^{13}C NMR ($CDCl_3$, 100 MHz): δ 147.8, 143.6, 140.5, 139.8, 138.1, 131.9, 118.0, 112.4, 53.5, 46.3, 34.4, 34.4, 34.3, 34.3, 33.3, 31.7, 29.7, 29.3, 28.2, 28.1, 25.6, 25.6, 22.7, 22.7, 14.1, 14.1, –8.4; HRMS (FD, $C_{47}H_{74}S_2Sn_2^{+\cdot}$): calcd, 942.3270; found, 942.3293.

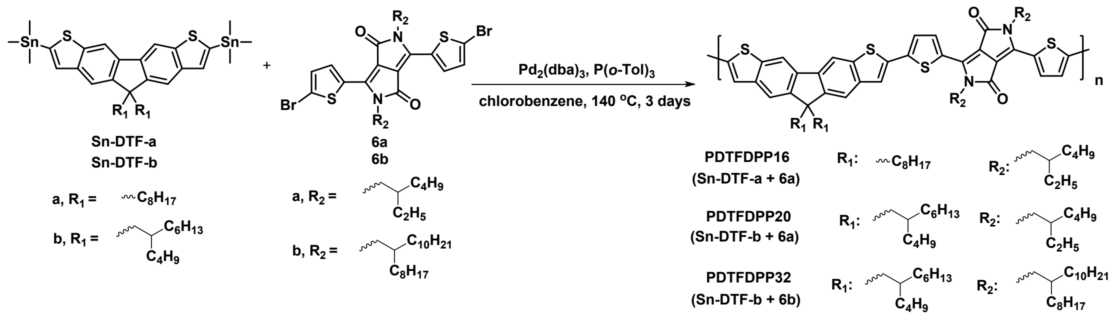

Compound **6a** and **6b** were synthesized as reported.[S3]

Synthesis of PDTFDPP16. To a 50 mL round-bottom flask were introduced Sn-DTF-a (190 mg, 0.23 mmol), **6a** (156 mg, 0.23 mmol), $Pd(PPh_3)_4$ (11 mg, 0.0095 mmol), tri(*o*-tolyl)phosphine (22 mg, 0.07 mmol), and dry chlorobenzene (5 mL). The mixture was degassed by bubbling nitrogen for 30 min at room temperature and refluxed for 3 days. Tributyl(thiophen-2-yl)stannane (41 mg, 0.11 mmol) was then added to the mixture and the heating was continued for 1 day. Subsequently, 2-bromothiophene (19.6 mg, 0.12 mmol) was added to the mixture and the heating was continued for another day. The resultant mixture was added into methanol dropwise. The precipitate was collected by filtration and washed by Soxhlet extraction with acetone (24 h) and hexane (24 h), sequentially. The crude product was re-dissolved in THF (200 mL). The Pd−thiol gel (Silicycle Inc.) was added to the above THF solution to remove the residual Pd catalyst

at 80 °C for 1 hour. After filtration and removal of the solvent under reduced pressure, the polymer was re-precipitated again from methanol/THF, collected by filtration, and dried under vacuum for 1 day to give a green black solid **PDTFDPP16** (153 mg, 65%, M_n = 4500, PDI = 1.2). ^{1}H NMR ($CDCl_3$, 400 MHz): δ 9.01-8.95 (br, 2 H), 7.74-7.40 (br, 6 H), 7.02-7.00 (br, 2H), 4.05 (br, 4H), 2.17-2.07 (br, 8 H), 1.96-0.79 (br, 56 H).

Synthesis of PDTFDPP20

To a 50 mL round-bottom flask were introduced **Sn-DTF-b** (213 mg, 0.23 mmol), **6a** (157 mg, 0.23 mmol), $Pd(PPh_3)_4$ (10.6 mg, 0.0092 mmol), tri(*o*-tolyl)phosphine (22.4 mg, 0.074 mmol), and dry chlorobenzene (6 mL). The mixture was degassed by bubbling nitrogen for 30 min at room temperature and refluxed for 3 days. Tributyl(thiophen-2-yl)stannane (42.9 mg, 0.12 mmol) was then added to the mixture and the heating was continued for 1 day. Subsequently, 2-bromothiophene (19.6 mg, 0.12 mmol) was added to the mixture and the heating was continued for another day. The resultant mixture was added into methanol dropwise. The precipitate was collected by filtration and washed by Soxhlet extraction with acetone (24 h) and hexane (24 h), sequentially. The crude product was re-dissolved in THF (200 mL). The Pd−thiol gel (Silicycle Inc.) was added to the above THF solution to remove the residual Pd catalyst at 80 °C for 1 h. After filtration and removal of the solvent under reduced pressure, the polymer was re-precipitated again from methanol/THF, collected by filtration, and dried under vacuum for 1 day to give a green black solid **PDTFDPP20** (150 mg, 58%, M_n = 68000, PDI = 2.1). ^{1}H NMR ($CDCl_3$, 400 MHz): δ 9.22-9.00 (br, 2 H), 7.79-7.74 (br, 2 H), 7.57-6.97 (br, 6 H), 4.05 (br, 4H), 1.96 (br, 8 H), 1.82-0.58 (br, 72 H).

Synthesis of PDTFDPP32.

To a 50 mL round-bottom flask were introduced **Sn-DTF-b** (183 mg, 0.19 mmol), **6b** (198 mg, 0.19 mmol), $Pd(PPh_3)_4$ (8.8 mg, 0.0076 mmol), tri(*o*-tolyl)phosphine (19 mg,

0.06 mmol), and dry chlorobenzene (6 mL). The mixture was degassed by bubbling nitrogen for 30 min at room temperature and refluxed for 3 days. Tributyl(thiophen-2-yl)stannane (37.8 mg, 0.095 mmol) was then added to the mixture and the heating was continued for 1 day. Subsequently, 2-bromothiophene (17.1 mg, 0.102 mmol) was added to the mixture and the heating was continued for another day. The resultant mixture was added into methanol dropwise. The precipitate was collected by filtration and washed by Soxhlet extraction with acetone (24 h) and hexane (24 h), sequentially. The crude product was re-dissolved in THF (200 mL). The Pd−thiol gel (Silicycle Inc.) was added to the above THF solution to remove the residual Pd catalyst at 80 °C for 1 h. After filtration and removal of the solvent under reduced pressure, the polymer was re-precipitated again from methanol/THF, collected by filtration, and dried under vacuum for 1 day to give a green black solid **PDTFDPP32** (210 mg, 73%, M_n = 32000, PDI = 1.8). ^{1}H NMR ($CDCl_3$, 400 MHz): δ 9.23-9.00 (br, 2 H), 7.78-7.32 (br, 6 H), 7.13-6.95 (br, 2H), 4.10 (br, 4H), 2.03 (br, 8 H), 1.38-0.64 (br, 120 H).

4. Thermogravimertic analysis (TGA)

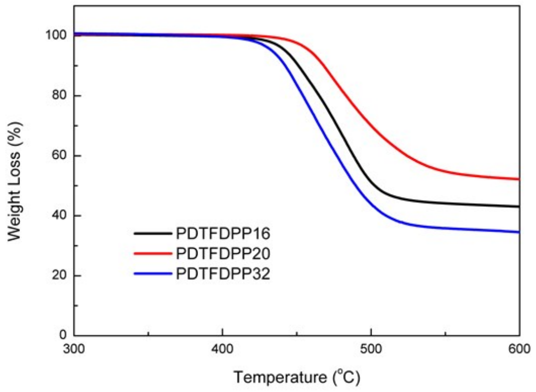

Figure S1. Thermogravimetric analyses of **PDTFDPP16**, **PDTFDPP20**, and **PDTFDPP32** at ramping rate of 10 °C/min.

5. Grazing Incidence X-ray Scattering (GIXS)

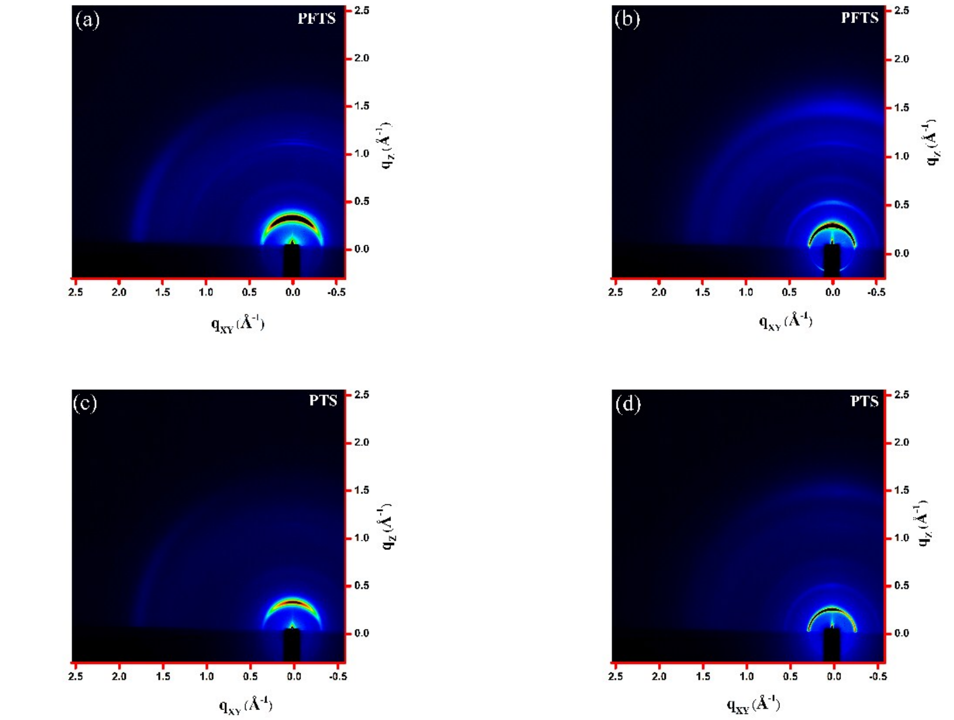

Figure S2. GIXS of **PDTFDPP16** (a and c) and **PDTFDPP20** (b and d) films on the PFTS and PTS-treated SAM surfaces, respectively.

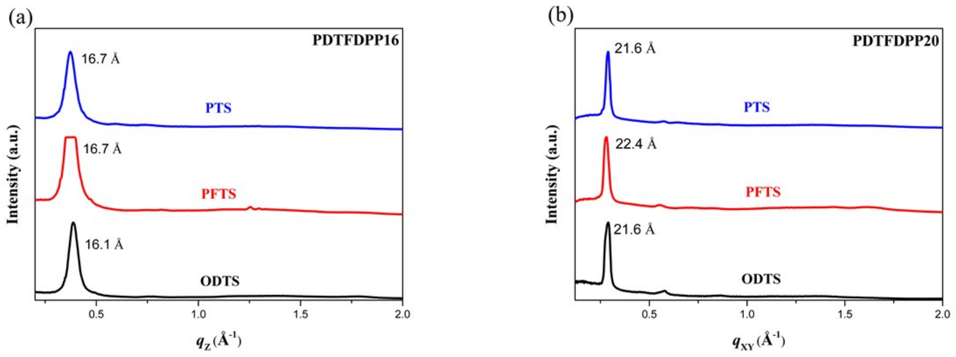

Figure S3. 1-Dimentional GIXS patterns of (a) **PDTFDPP16** in q_z and (b) **PDTFDPP20** in q_{xy} direction on PTS, PFTS and ODTS-treated SAM surfaces.

Table S1. Additional GIXS data

	substrate	lamellar L_c (nm)	π-stacking L_c (nm)	R_{Lc}	thin-film stacking
**P3HT**	ODTS[a]/SiO_2	6.40[c]	3.16[c]	2.03	edge-on
**PBTTT-C12**[S3]	OTS[b]/SiO_2	12.57	6.61	1.90	edge-on
**PTDP3T**[S4] (M_n = 8.4 kg/mol)	Si/SiO_2	23.80	5.20	4.58	face-on
**PDPP3F-BO**[S5]	OTS/SiO_2	11.00	1.40	7.86	face-on
**PDPP3F-C16**[S5]	OTS/SiO_2	9.70	3.90	2.49	edge-on

[a] ODTS = octadecyltrichlorosilane

[b] OTS = octyltrichlorosilane

[c] Measurement was carried out by ourselves

6. OFET Data

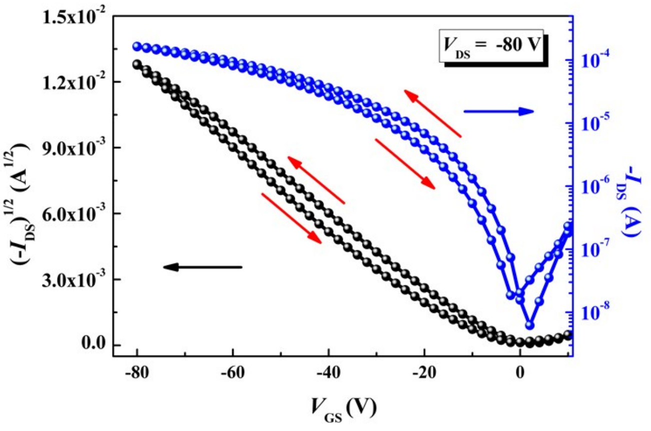

Figure S4. The transfer curves for **PDTFDPP20** with the forward (20 V to -80 V) and backward (-80 V to 20 V) sweeping of bias voltage.

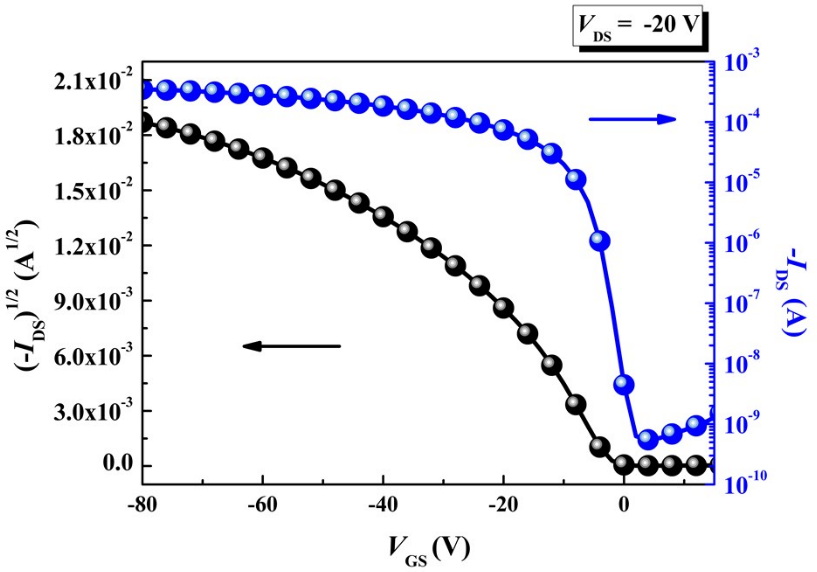

Figure S5. Typical transfer curves of **PDTFDPP20** measured in the linear regime with V_{DS} of -20 V.

7. Polymer Information

Table S2. Amount of DTF monomers used for polymerization and the molecular weights of **PDTFDPP20** and **PDTFDPP32** obtained in three different batches.

	Amount of DTF monomer	M_n (g/mol)
**PDTFDPP20**	200 mg	54000
	210 mg	60000
	200 mg	68000
**PDTFDPP32**	205 mg	28000
	200 mg	29000
	190 mg	32000

8. Atomic Force Microscopy

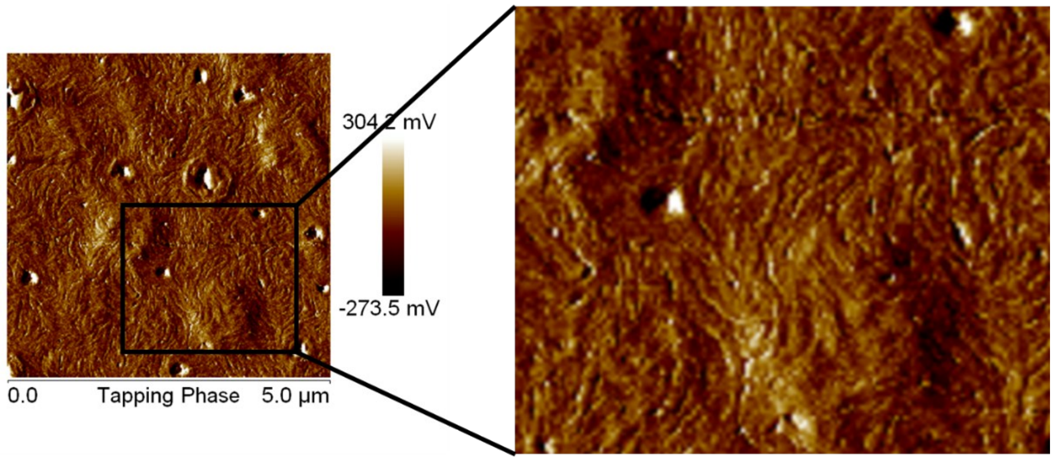

Figure S6. Enlarged AFM phase image of Figure 10 (e) for **PDTFDPP20**.

9. ^{1}H and ^{13}C NMR spectra

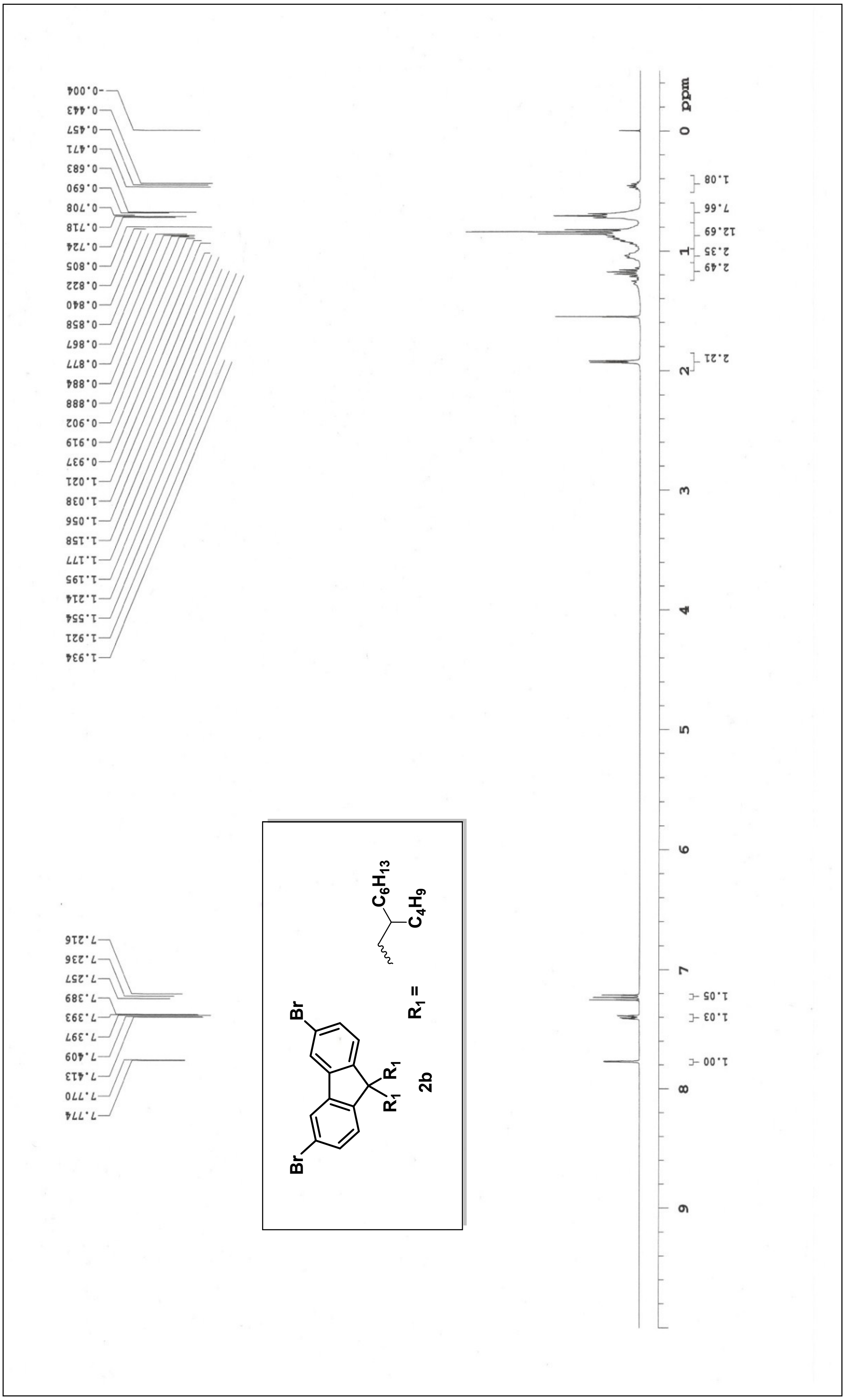
ppm
Br
Br
2b
R_1 =

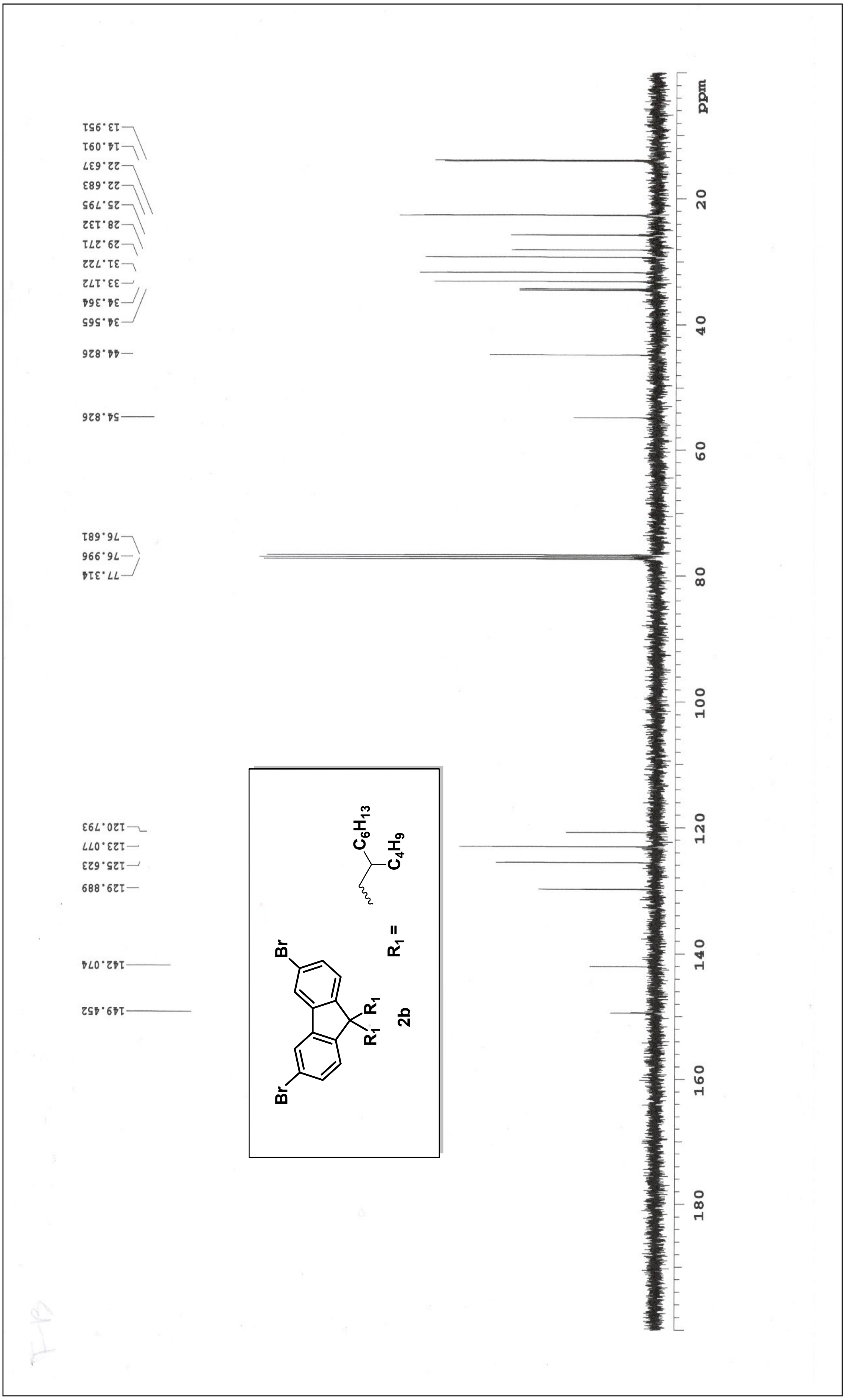
13.951
14.091
22.637
22.683
25.795
28.132
29.271
31.722
33.172
34.364
34.565
44.826
54.826
76.681
76.996
77.314
120.793
123.077
125.623
129.889
142.074
149.452
ppm
20
40
60
80
100
120
140
160
180
Br
Br
R1
R1
2b
R1 =
C6H13
C4H9

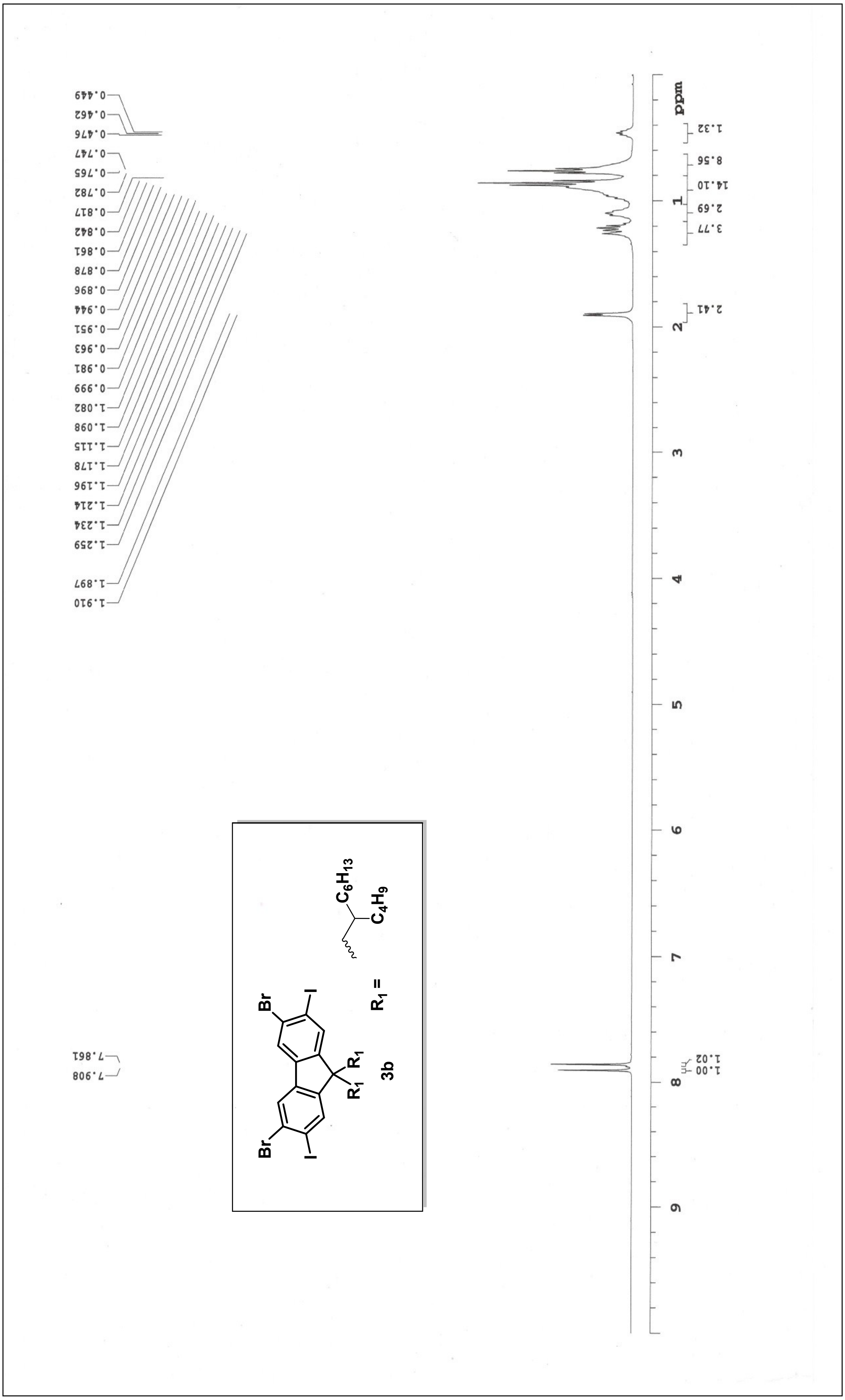
0.449
0.462
0.476
0.747
0.765
0.782
0.817
0.842
0.861
0.878
0.896
0.944
0.951
0.963
0.981
0.999
1.082
1.098
1.115
1.178
1.196
1.214
1.234
1.259
1.897
1.910
7.861
7.908
1.32
8.56
14.10
2.69
3.77
2.41
1.02
1.00
ppm
Br
I
R1
3b
R1 =
C6H13
C4H9

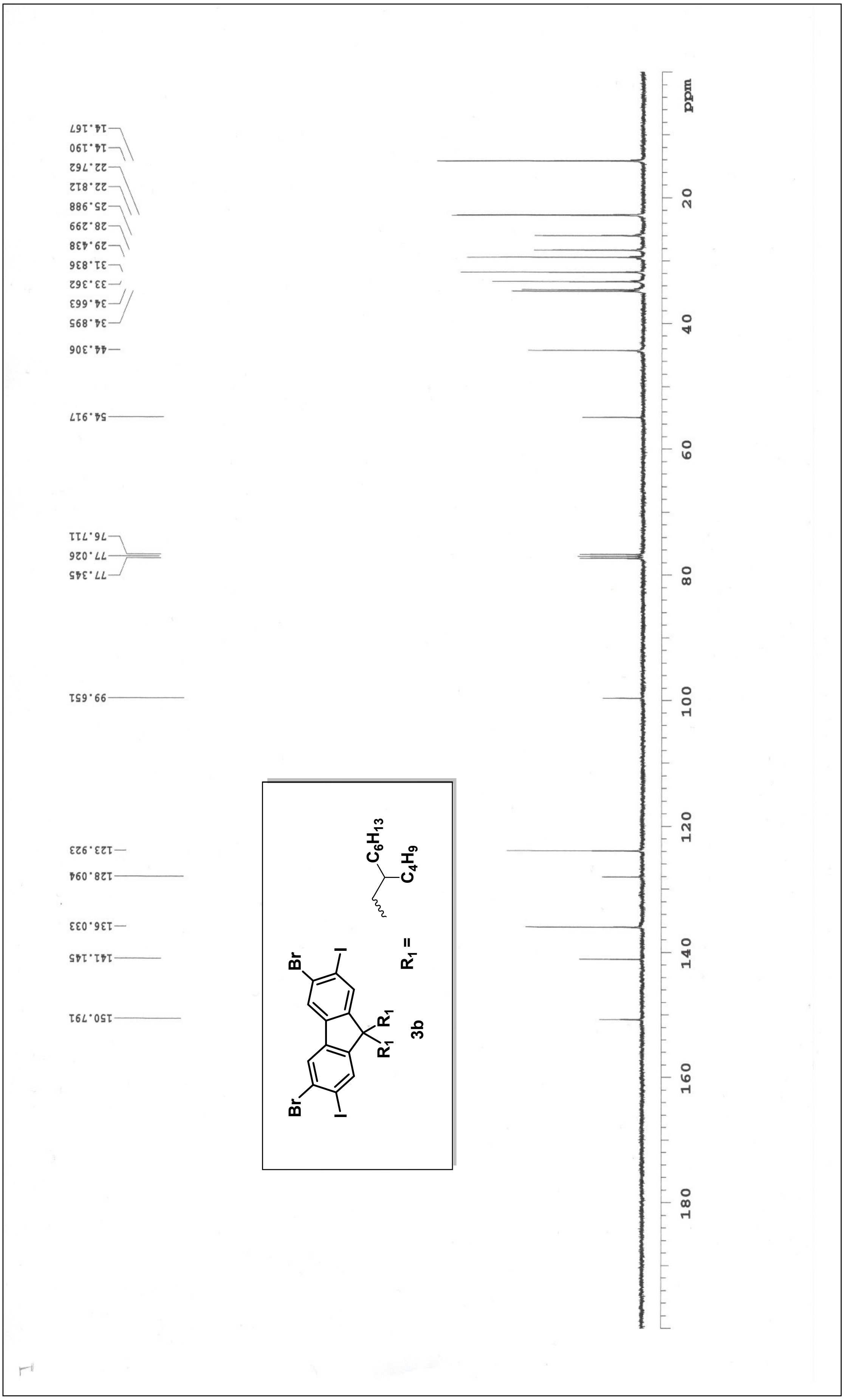
14.167
14.190
22.762
22.812
25.988
28.299
29.438
31.836
33.362
34.663
34.895
44.306
54.917
76.711
77.026
77.345
99.651
123.923
128.094
136.033
141.145
150.791
Br
I
R1
3b
R1 =
C6H13
C4H9
ppm
20
40
60
80
100
120
140
160
180

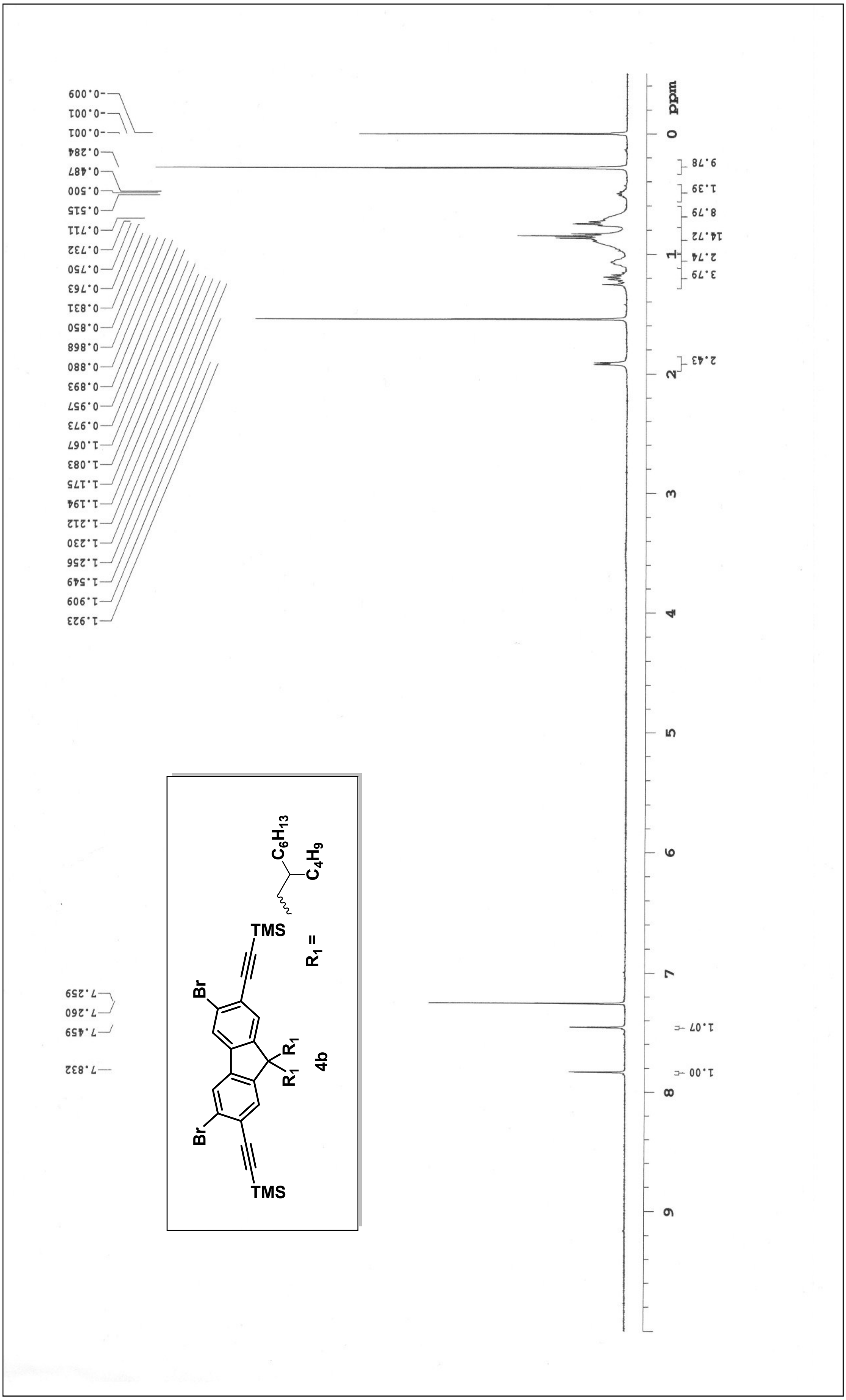
0.009
0.001
0.001
0.284
0.487
0.500
0.515
0.711
0.732
0.750
0.763
0.831
0.850
0.868
0.880
0.893
0.957
0.973
1.067
1.083
1.175
1.194
1.212
1.230
1.256
1.549
1.909
1.923
7.259
7.260
7.459
7.832
9.78
1.39
8.79
14.72
2.74
3.79
2.43
1.07
1.00
ppm
Br
Br
TMS
TMS
R1
R1
4b
R1 =
C6H13
C4H9

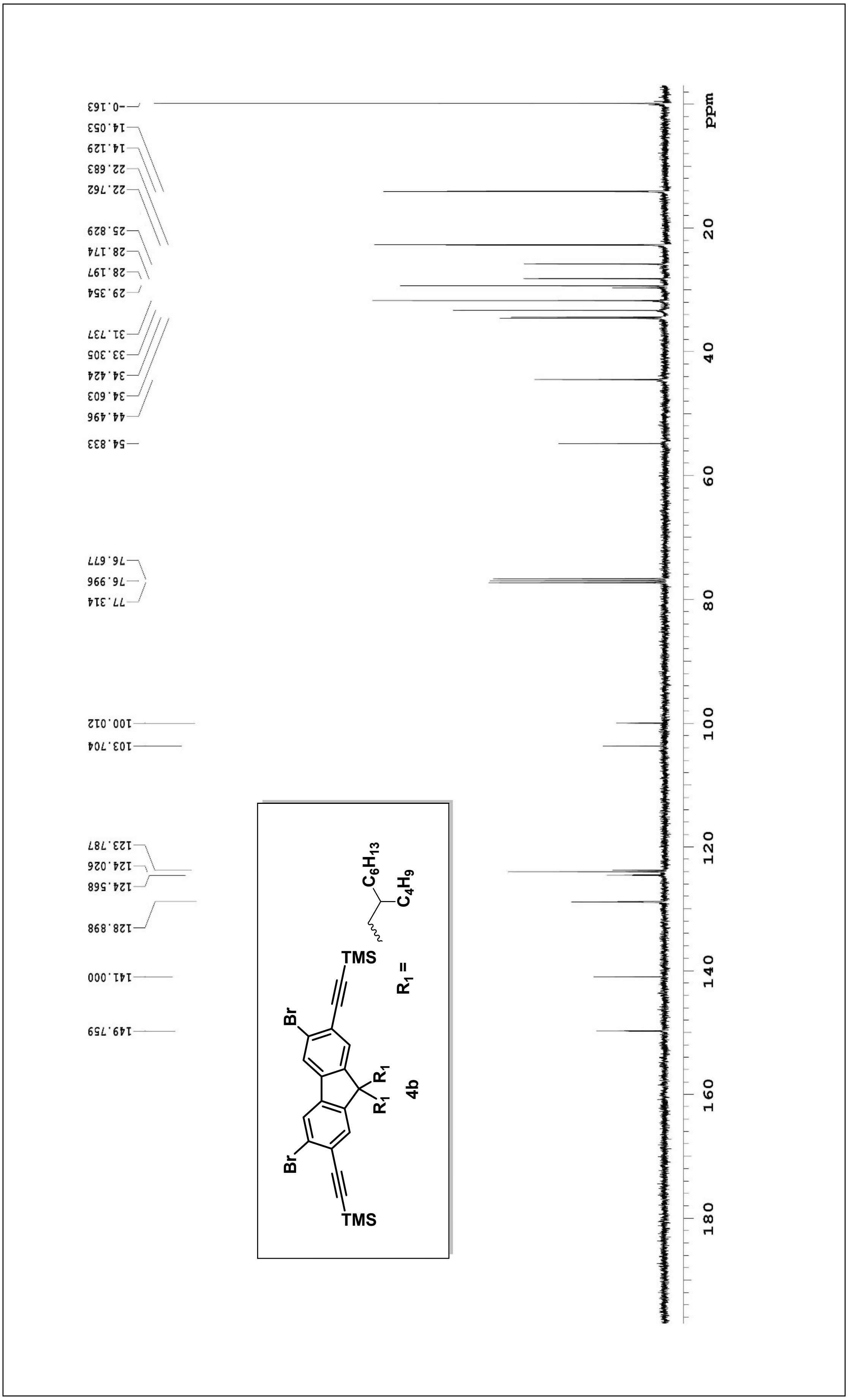
-0.163
14.053
14.129
22.683
22.762
25.829
28.174
28.197
29.354
31.737
33.305
34.424
34.603
44.496
54.833
76.677
76.996
77.314
100.012
103.704
123.787
124.026
124.568
128.898
141.000
149.759
ppm
Br
TMS
R_1 =
C_6H_{13}
C_4H_9
4b

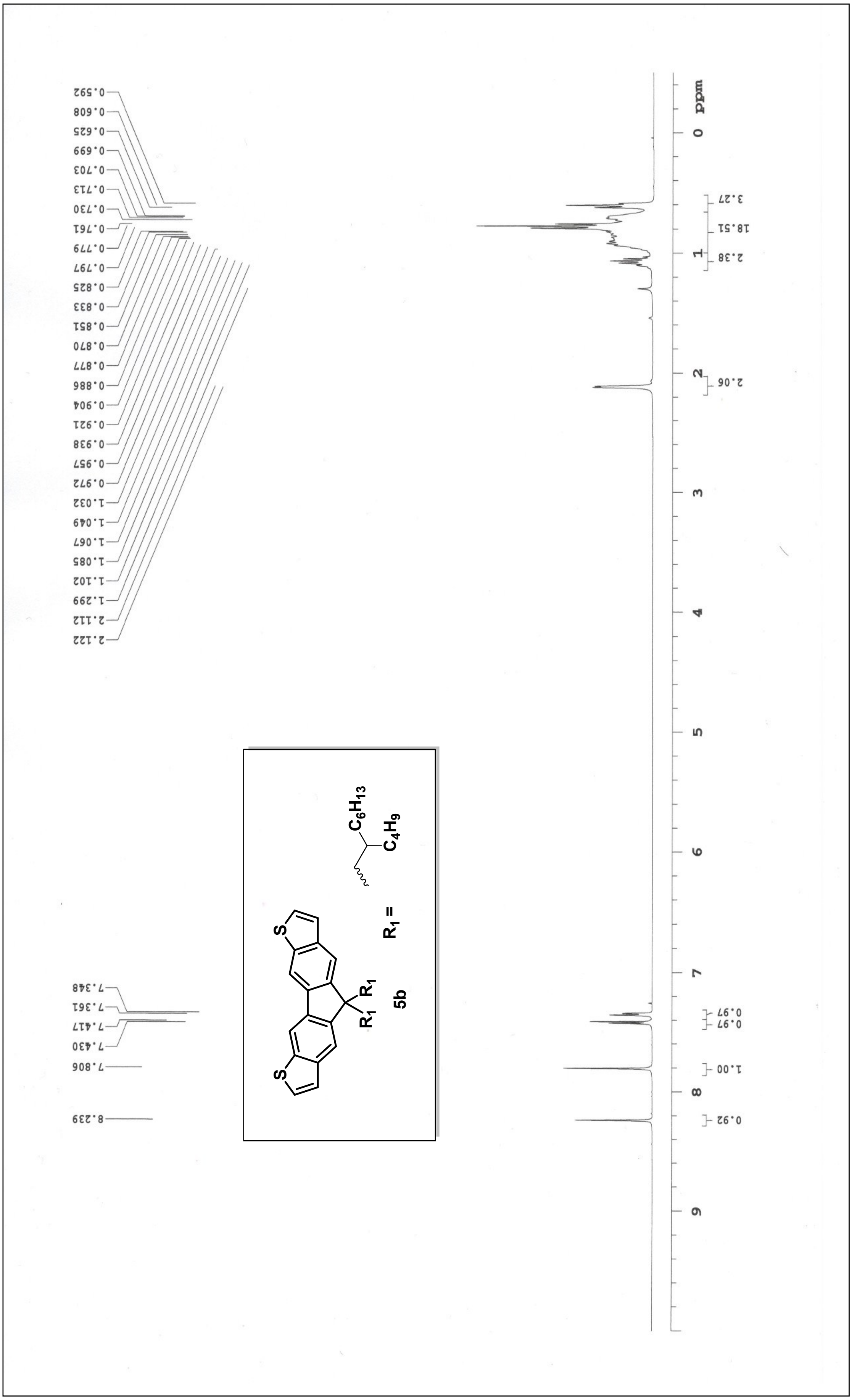
0.592
0.608
0.625
0.699
0.703
0.713
0.730
0.761
0.779
0.797
0.825
0.833
0.851
0.870
0.877
0.886
0.904
0.921
0.938
0.957
0.972
1.032
1.049
1.067
1.085
1.102
1.299
2.112
2.122
7.348
7.361
7.417
7.430
7.806
8.239
3.27
18.51
2.38
2.06
0.97
0.97
1.00
0.92
ppm
5b
R1 =
C6H13
C4H9

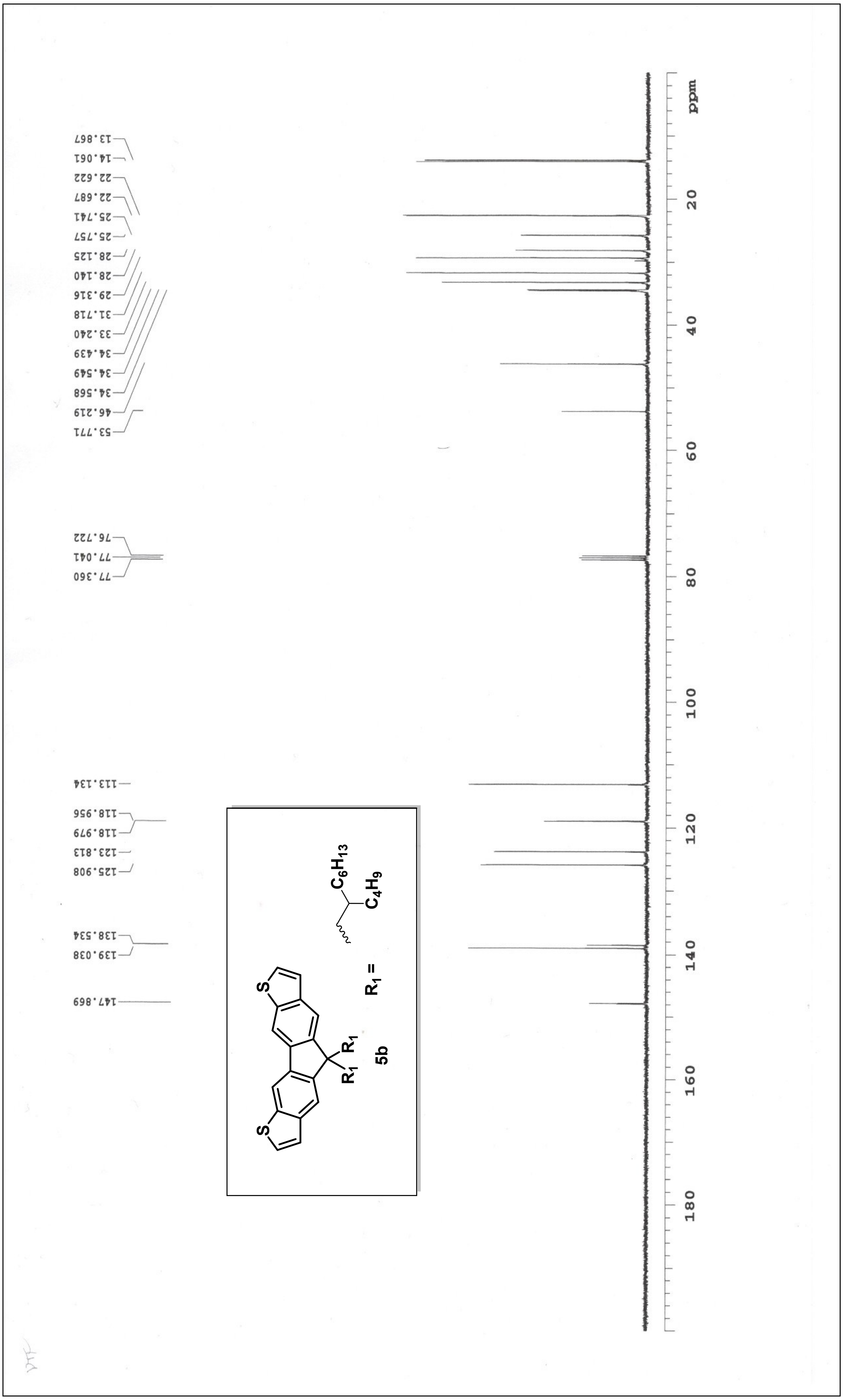
13.867
14.061
22.622
22.687
25.741
25.757
28.125
28.140
29.316
31.718
33.240
34.439
34.549
34.568
46.219
53.771
76.722
77.041
77.360
113.134
118.956
118.979
123.813
125.908
138.534
139.038
147.869
S
S
R1
R1
5b
R1 =
C6H13
C4H9
ppm
20
40
60
80
100
120
140
160
180

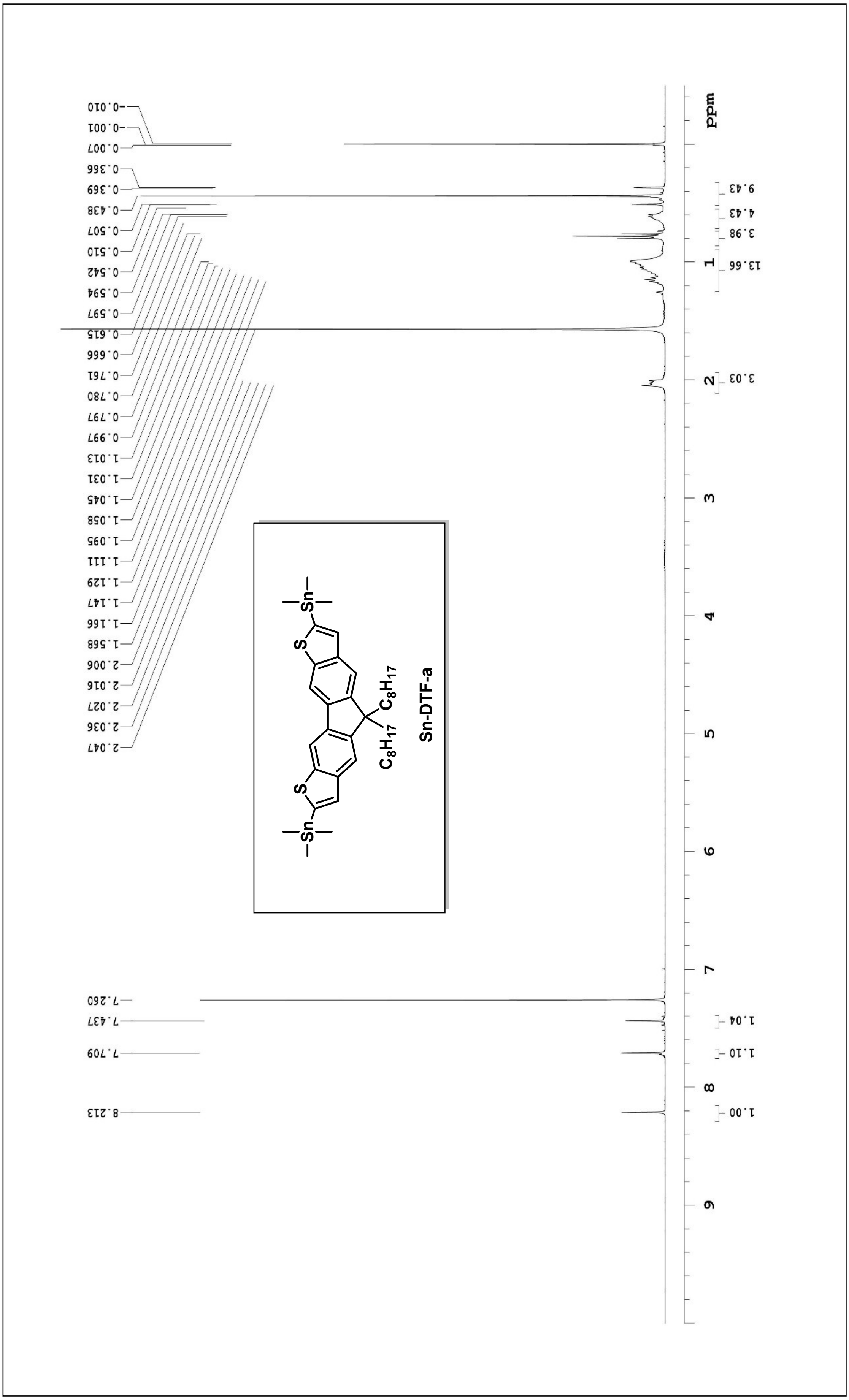
-0.010
-0.001
0.007
0.366
0.369
0.438
0.507
0.510
0.542
0.594
0.597
0.615
0.666
0.761
0.780
0.797
0.997
1.013
1.031
1.045
1.058
1.095
1.111
1.129
1.147
1.166
1.568
2.006
2.016
2.027
2.036
2.047
7.260
7.437
7.709
8.213
9.43
4.43
3.98
13.66
3.03
1.04
1.10
1.00
ppm
Sn
S
C8H17
Sn-DTF-a

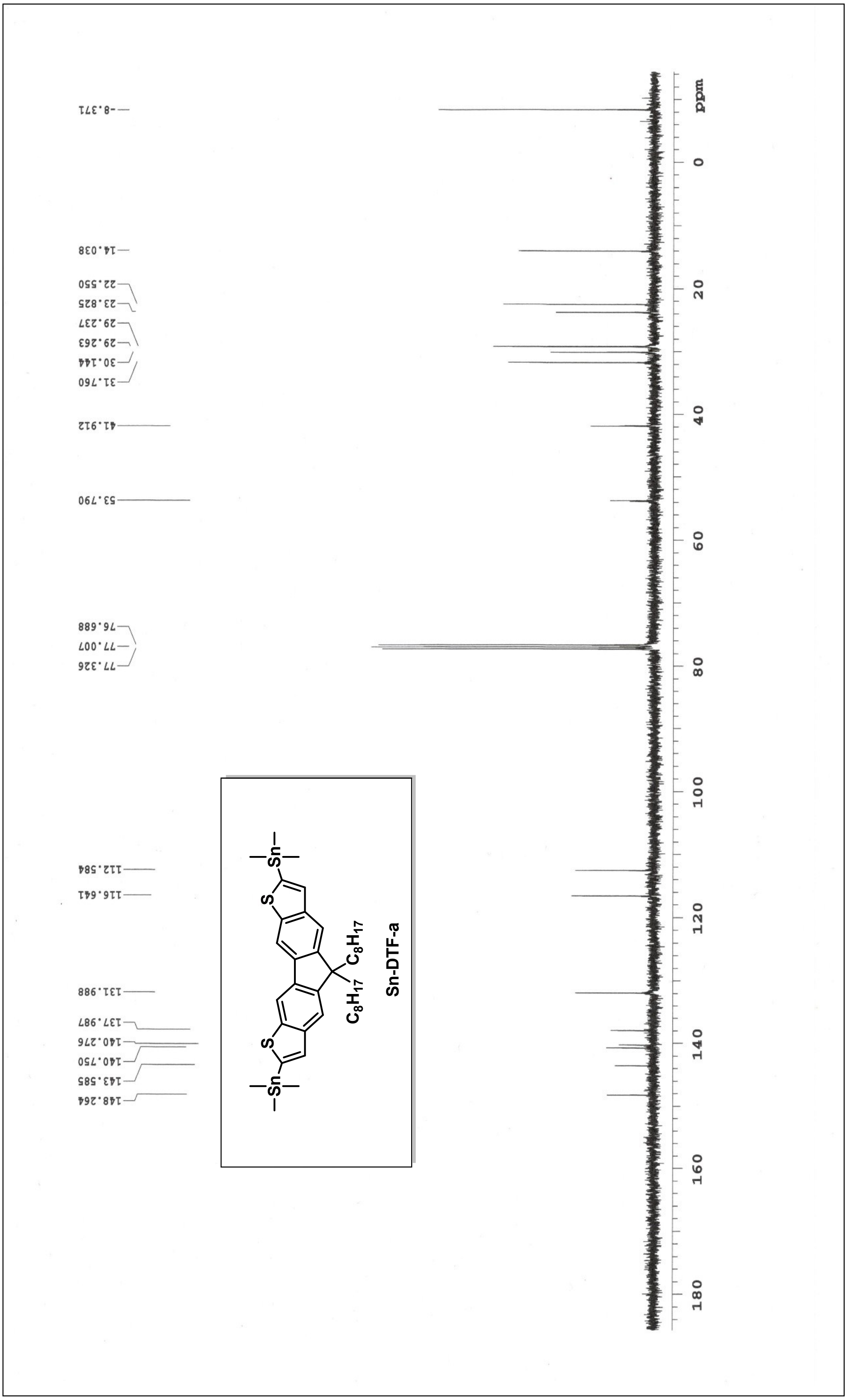
-8.371
14.038
22.550
23.825
29.237
29.263
30.144
31.760
41.912
53.790
76.688
77.007
77.326
112.584
116.641
131.988
137.987
140.276
140.750
143.585
148.264
ppm
Sn-DTF-a

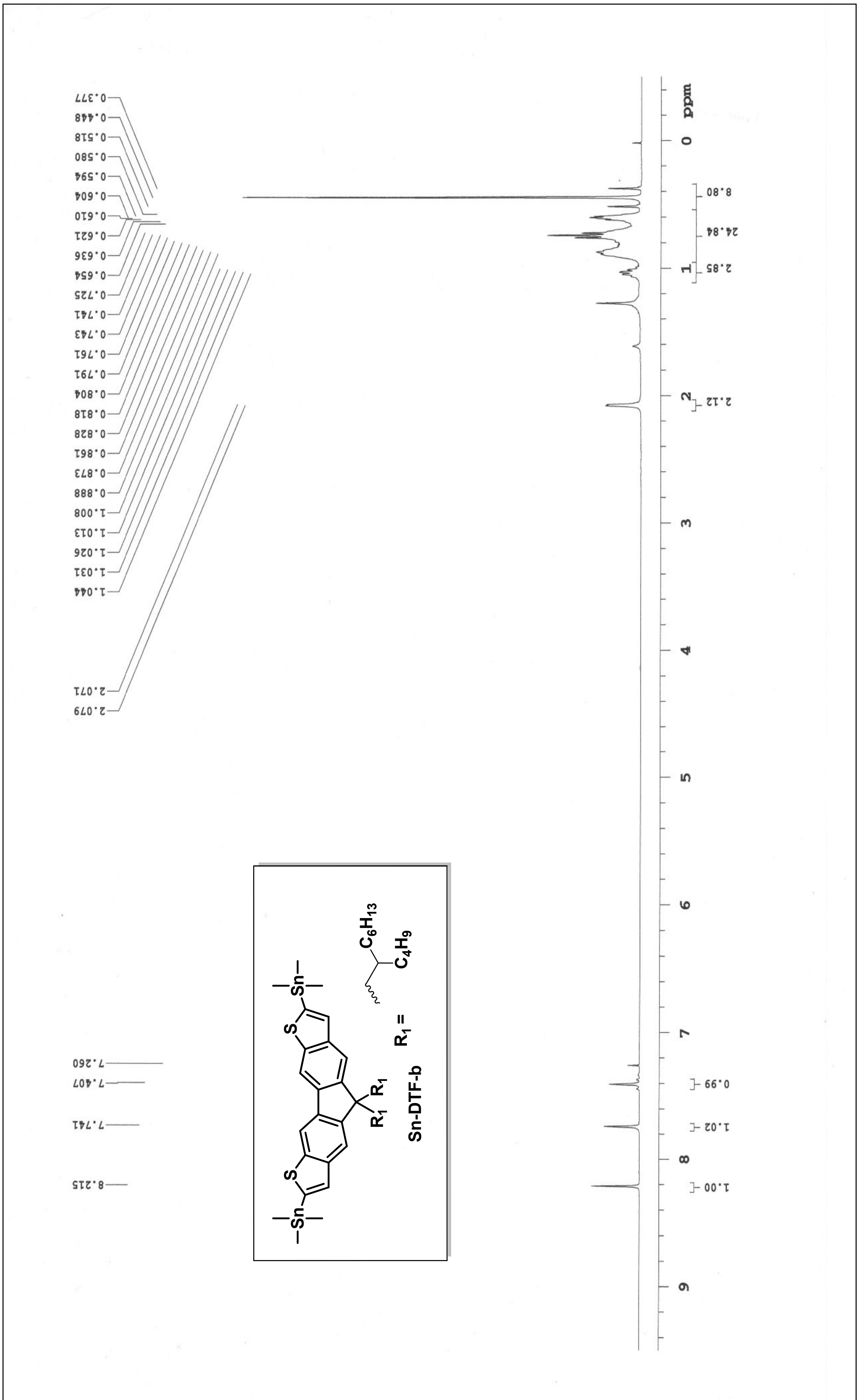
Sn-DTF-b
R1 =
C6H13
C4H9
ppm

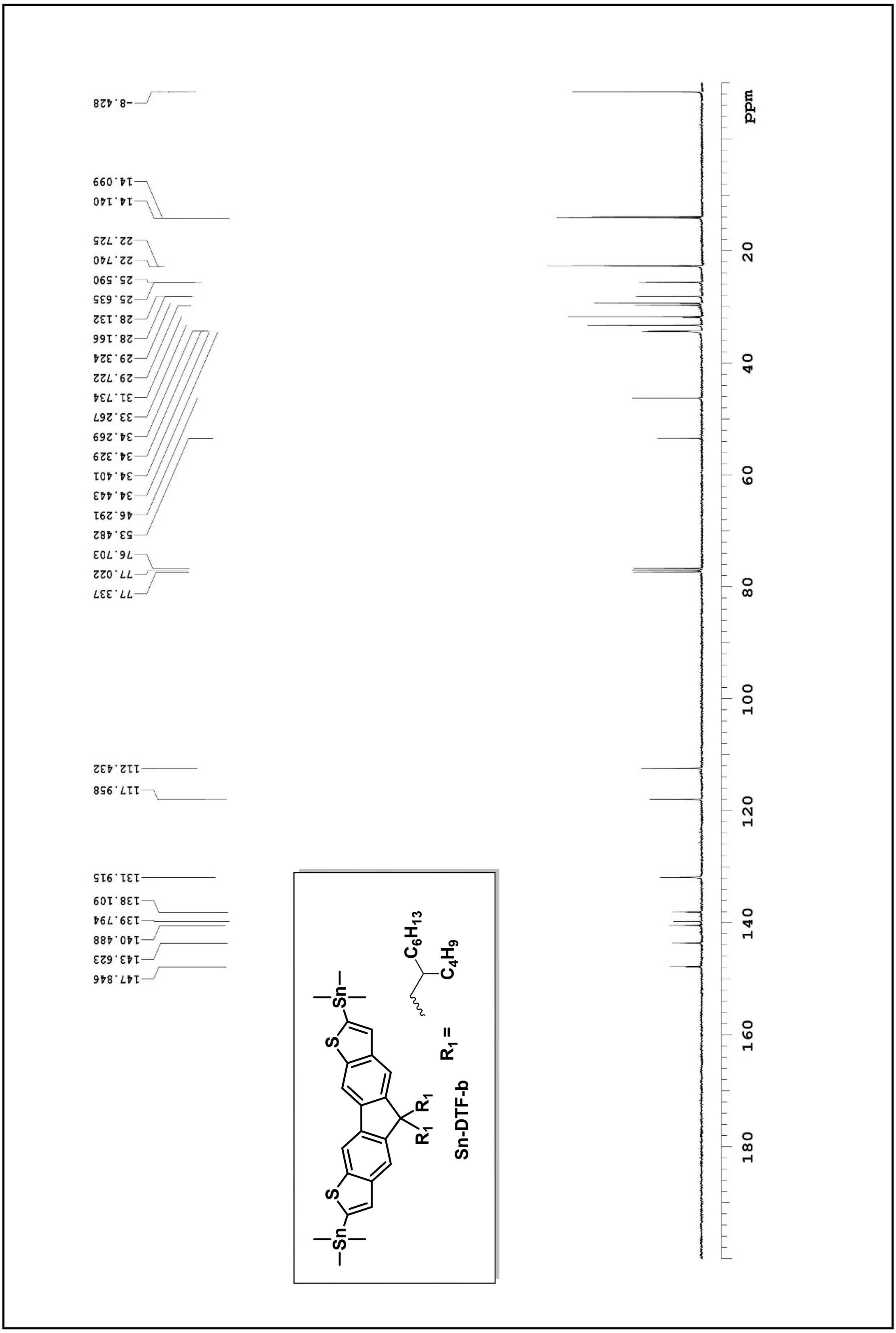
-8.428
14.099
14.140
22.725
22.740
25.590
25.635
28.132
28.166
29.324
29.722
31.734
33.267
34.269
34.329
34.401
34.443
46.291
53.482
76.703
77.022
77.337
112.432
117.958
131.915
138.109
139.794
140.488
143.623
147.846
Sn-DTF-b
R_1 =
C_6H_{13}
C_4H_9
ppm
20
40
60
80
100
120
140
160
180

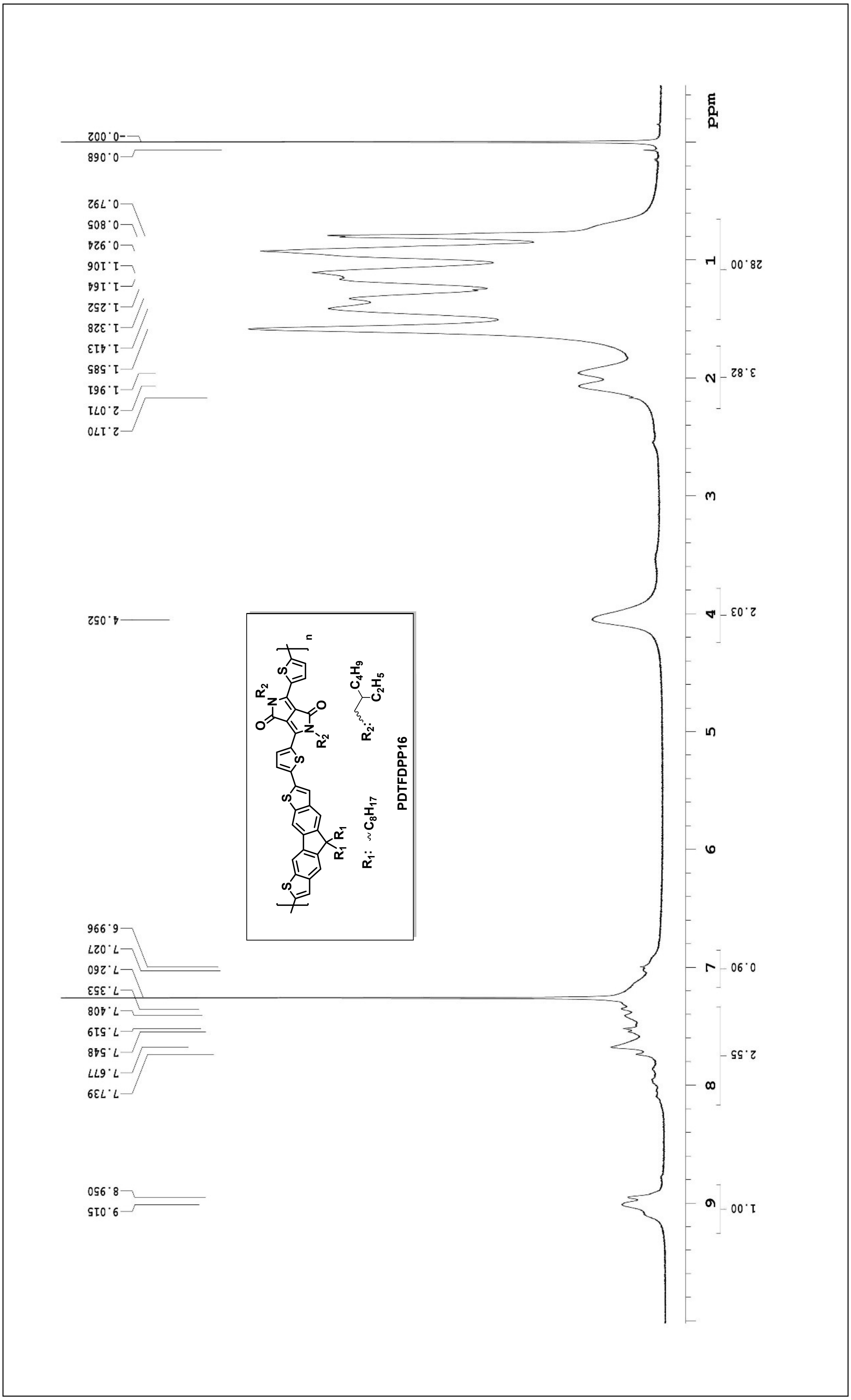

-0.002
0.068
0.792
0.805
0.924
1.106
1.164
1.252
1.328
1.413
1.585
1.961
2.071
2.170
4.052
6.996
7.027
7.260
7.353
7.408
7.519
7.548
7.677
7.739
8.950
9.015
28.00
3.82
2.03
0.90
2.55
1.00
ppm
PDTFDPP16
R_1: C_8H_{17}
R_2: C_4H_9 C_2H_5

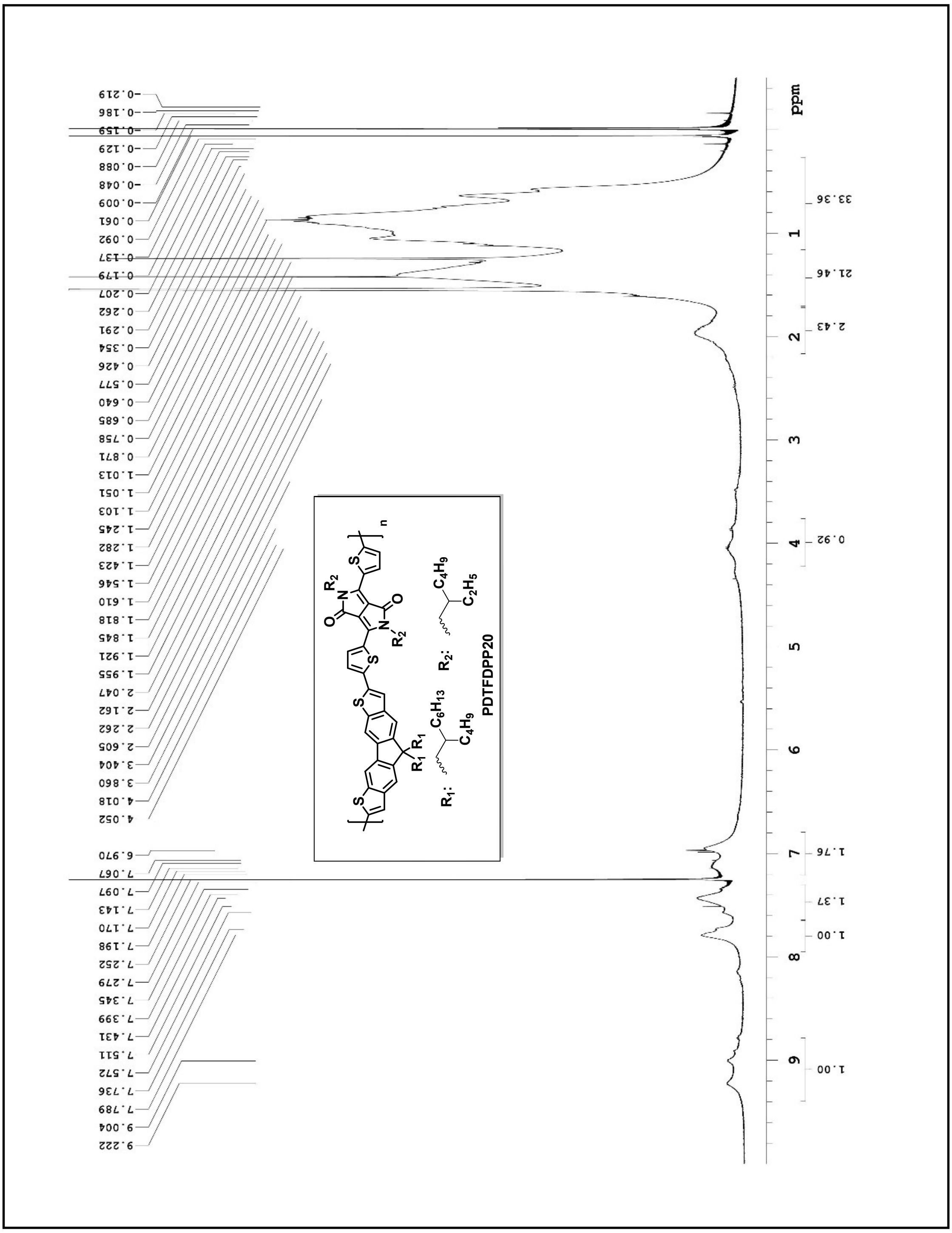
PDTFDPP20
ppm

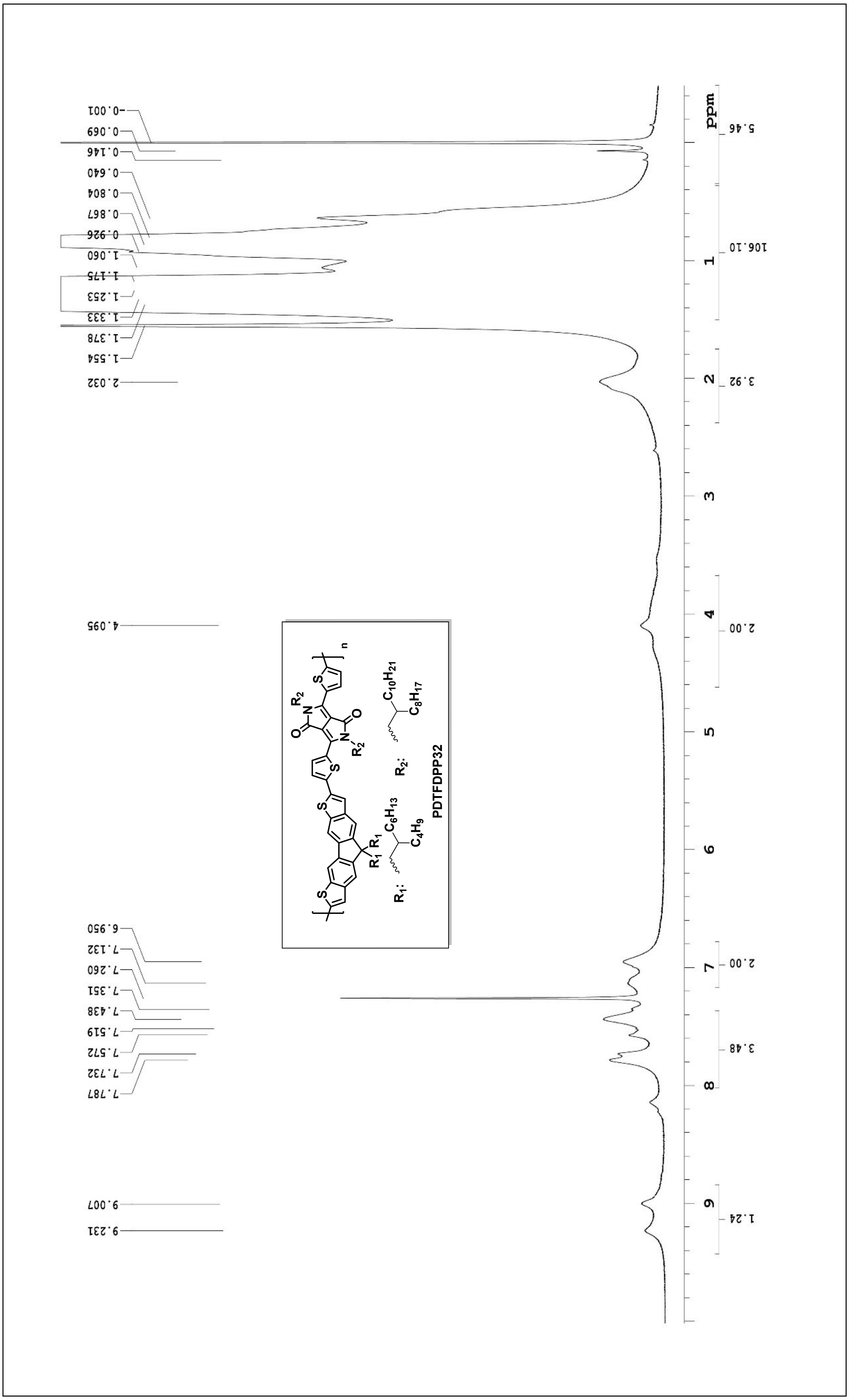

-0.001
0.069
0.146
0.640
0.804
0.867
0.926
1.060
1.175
1.253
1.333
1.378
1.554
2.032
4.095
6.950
7.132
7.260
7.351
7.438
7.519
7.572
7.732
7.787
9.007
9.231
ppm
5.46
106.10
3.92
2.00
2.00
3.48
1.24
PDTFDPP32
R1: C6H13 C4H9
R2: C10H21 C8H17

10. Computational details.

Two layer ONIOM calculations were performed with the Gaussian09 suite[S6] employing B3LYP/6-31G(d) to conjugated systems and UFF to alkyl side chains. In our computation, we set up the dimeric model compounds, 2DTFDPP16, 2DTFDPP20, and 2DTFDPP32 for simulating **PDTFDPP16**, **PDTFDPP20**, and **PDTFDPP32**, respectively.

Cartesian coordinates (Å) of the Gaussian ONIOM (B3LYP/6-31G(d):UFF) optimized

2DTFDPP16

C	-5.134240082	-3.429306561	-0.805969861
C	-5.735002245	-3.658238684	0.430414033
C	-4.957023398	-3.845216904	1.606704964
C	-3.576018031	-3.803623064	1.556893733
C	-2.944076756	-3.56897773	0.317378944
C	-3.740998258	-3.387074064	-0.846003101
H	-5.726339055	-3.288047762	-1.70563338
H	-2.971847687	-3.946377176	2.44954983
C	-5.882006797	-4.076137462	2.810713496
C	-7.153161842	-3.750746134	0.775802635
C	-7.276353388	-3.996274675	2.172134777
C	-8.276397719	-3.634380416	-0.040653466
C	-9.526687981	-3.765539085	0.562811187
H	-8.184381064	-3.448138407	-1.106826801
C	-9.673117352	-4.009665746	1.95586262
C	-8.519885788	-4.126237542	2.761292574
H	-8.625325676	-4.314507006	3.827054817
S	-11.09090504	-3.656510465	-0.229195725
C	-11.93150269	-3.951717788	1.30423543
C	-11.04586931	-4.111470696	2.340656541
H	-11.36070041	-4.31477201	3.359318012
S	-2.738239728	-3.106962412	-2.261454933
C	-1.260781923	-3.253289716	-1.29174749
C	-1.545340073	-3.489013467	0.028619931
H	-0.775425292	-3.627137267	0.781024563
C	-13.37249262	-3.998219058	1.337627156
C	-14.26870652	-4.043424606	0.283276857
S	-14.23162067	-3.985454177	2.859367285
C	-15.61780812	-4.08876923	0.679548119
H	-13.95369709	-4.056378472	-0.754500502
C	-15.79851299	-4.09459458	2.057781456
H	-16.45285506	-4.098814491	-0.011087116
C	-17.08594892	-4.112290426	2.711335281
C	-18.29512041	-3.777359682	2.105120645

N	-17.38888794	-4.410372602	4.056744239
C	-19.34188913	-3.813676978	3.071875894
C	-18.78619587	-4.216497396	4.35453839
O	-19.29363518	-4.370159966	5.453033476
C	-18.86757155	-3.41587238	0.815632
N	-20.28044674	-3.31085889	1.105601026
C	-20.55398702	-3.517800557	2.47743049
O	-18.38143433	-3.247974823	-0.295140362
C	-21.88205073	-3.527254024	3.0521996
C	-23.08026578	-3.902788067	2.466604607
S	-22.14185357	-3.050752223	4.719651437
C	-24.19081509	-3.793078677	3.340343062
H	-23.15096195	-4.299643834	1.460333979
C	-23.83846583	-3.332639271	4.583906131
H	-25.20529042	-4.055913197	3.062039803
H	-24.48150408	-3.160769914	5.437080512
C	19.07534336	-1.842692141	2.056578655
C	18.03714172	-2.495646209	2.715299082
C	18.25404753	-3.171670556	3.947561847
C	19.50885895	-3.19918712	4.529033816
C	20.57684029	-2.545130397	3.881959541
C	20.33828616	-1.875951561	2.651391959
H	18.91244622	-1.325289374	1.115345823
H	19.68403812	-3.713333871	5.471256191
C	16.93849592	-3.793981845	4.438711498
C	16.62846503	-2.627714216	2.339420624
C	15.95184144	-3.38797357	3.333950882
C	15.95589276	-2.135639887	1.22301526
C	14.59344078	-2.415357579	1.117250943
H	16.47057001	-1.555977274	0.462141846
C	13.89778136	-3.172489264	2.09898506
C	14.6018146	-3.661527261	3.220229569
H	14.07560634	-4.242215867	3.974124443
S	13.51613216	-1.913936356	-0.176803676
C	12.14474152	-2.731631458	0.595299254
C	12.51608955	-3.334038416	1.771478004
H	11.82440618	-3.898328235	2.388983819
S	21.81339501	-1.13855689	2.039881044

C	22.7147066	-1.728661748	3.420882937
C	21.95236787	-2.439054521	4.29200724
H	22.33953734	-2.881288303	5.203935638
C	10.8452109	-2.704760917	-0.028517681
C	10.49463921	-2.276275877	-1.298015109
S	9.428819673	-3.252992304	0.834902006
C	9.124669644	-2.402977138	-1.587802874
H	11.21847846	-1.891354419	-2.008142542
C	8.376823437	-2.947528137	-0.548449201
H	8.670400713	-2.097663076	-2.523887334
C	6.952433536	-3.170105726	-0.604798078
C	6.090981838	-2.628026358	-1.560998531
N	6.141651103	-3.929856927	0.265255623
C	4.755394648	-3.023809802	-1.280820694
C	4.747386999	-3.868851941	-0.098766813
O	3.845625997	-4.423413959	0.507191396
C	6.088802302	-1.837449704	-2.781750214
N	4.700256184	-1.805428497	-3.152147405
C	3.903785758	-2.548847581	-2.261075253
O	6.983055041	-1.29770923	-3.424920053
C	2.481542406	-2.788403846	-2.398611009
C	1.721885875	-3.024726247	-3.533907751
S	1.462035967	-2.795377764	-0.973774544
C	0.349362751	-3.207948082	-3.266594356
H	2.141007732	-3.134399576	-4.526938974
C	0.031743471	-3.113849378	-1.922735949
H	-0.383679851	-3.437832587	-4.032109923
H	23.77084612	-1.504926215	3.496196348
C	-5.63496305	-5.457143676	3.456522183
H	-4.60571403	-5.48344388	3.878751889
H	-6.344161625	-5.596109955	4.302727144
C	-21.04137053	-2.332274519	0.33792437
C	-16.48402286	-4.952234012	5.047849396
H	-20.65305912	-2.278198426	-0.701907438
H	-22.0826179	-2.694907435	0.23308819
H	-15.62486396	-4.262293413	5.166918353
H	-16.97195205	-4.97521799	6.045661089
C	-21.0478677	-0.910344716	0.971195218

H	-21.30334548	-1.011748861	2.047722754
C	-22.18029408	-0.042759126	0.358375433
C	-19.64917428	-0.24606083	0.898546782
H	-23.14605662	-0.581132474	0.482178971
H	-22.28127032	0.897504588	0.941687879
C	-21.9886787	0.298638793	-1.130083822
H	-21.93757555	-0.635835646	-1.72782006
H	-21.04343075	0.863803862	-1.2707834
C	-23.15279893	1.151734809	-1.647395883
H	-24.10873012	0.597776261	-1.522109591
H	-23.21393533	2.096201918	-1.063741102
C	-22.97225099	1.495499116	-3.123188003
H	-22.93344205	0.568115903	-3.733539129
H	-22.03524894	2.073125459	-3.273068057
C	4.206515133	-0.909389964	-4.179241337
C	6.598589034	-4.842290692	1.291469255
H	5.05041815	-0.470155003	-4.755072825
H	3.628806992	-1.497688275	-4.914204716
H	7.246625418	-4.291449887	2.000812027
H	5.746021486	-5.19658209	1.911535004
C	3.356240526	0.245278814	-3.585158511
C	7.325879358	-6.073267711	0.689607714
H	2.58597105	-0.184061151	-2.910640673
H	8.132725073	-5.706964011	0.018531266
C	2.629614091	1.01881556	-4.7151843
C	4.249340807	1.198526193	-2.749176643
C	8.005584153	-6.875834723	1.825867982
C	6.341368236	-6.965663716	-0.123408913
H	5.000247518	0.610151653	-2.181116681
H	4.809242353	1.8821764	-3.42513695
C	3.444109476	2.015093748	-1.724545084
H	2.892158253	1.319628809	-1.054221583
H	2.705875187	2.662030916	-2.242408959
H	2.264743085	1.997432951	-4.341913716
H	3.33342652	1.231879677	-5.549365538
H	8.478169683	-6.179703133	2.553144072
H	7.248628918	-7.472734308	2.380392484
C	9.111790525	-7.798728218	1.309088424

H	8.710281626	-8.557482041	0.607065851
H	9.897306053	-7.205882558	0.793655177
H	6.564151061	-8.040050095	0.038952228
H	5.298870697	-6.816165246	0.232299479
C	6.420894986	-6.692288856	-1.636388515
H	7.401623678	-7.041784162	-2.027175675
H	6.362432454	-5.602201942	-1.824490311
C	16.51847724	-3.219265132	5.809405657
H	15.54854982	-3.67240974	6.113115123
H	17.27367469	-3.509481234	6.573448117
C	-16.0057306	-6.384093184	4.685870464
H	-15.67621227	-6.388803904	3.624991445
C	-14.74463667	-6.767863877	5.500171066
H	-13.93452664	-6.038971052	5.276492096
C	-17.12887667	-7.445499612	4.845598674
H	-17.80886369	-7.176484755	5.682756729
C	-5.79559659	-6.641346789	2.483127621
H	-5.076868452	-6.536553053	1.641749033
H	-6.826454175	-6.649292259	2.067523546
C	-5.536853489	-7.974615762	3.193865524
H	-4.50445103	-7.976245953	3.608177712
H	-6.255984051	-8.088659418	4.034916485
C	-5.696891691	-9.153483353	2.2266713
H	-4.977837052	-9.039280485	1.385503558
H	-6.729206233	-9.151421867	1.811976574
C	-5.438578179	-10.48744784	2.936844887
H	-4.406237303	-10.48952441	3.351426661
H	-6.157678305	-10.60160089	3.777961733
C	-5.598736988	-11.66613629	1.969532174
H	-4.879586196	-11.55224005	1.128390748
H	-6.631094312	-11.66428619	1.554918035
C	-5.340413598	-12.99965728	2.680036418
H	-4.308531526	-13.0102972	3.094122359
H	-6.058354505	-13.1222407	3.520212537
C	-5.498876321	-14.17537802	1.720385888
H	-4.773864451	-14.09044784	0.882989042
H	-5.306283042	-15.12556691	2.261696514
H	-6.531179416	-14.202865	1.310894837

C	-5.690701602	-2.98534419	3.887259867
H	-6.399242837	-3.171306461	4.724943366
H	-4.660609697	-3.058830213	4.301792337
C	-5.909418146	-1.549947208	3.370038845
H	-5.193699757	-1.333481106	2.54763898
H	-6.942990141	-1.446225487	2.974518194
C	-5.701779562	-0.525254383	4.49100894
H	-4.667404471	-0.620294528	4.889194618
H	-6.419008873	-0.73203956	5.315831139
C	-5.917945747	0.903084983	3.977250919
H	-6.95238173	0.998084886	3.579052427
H	-5.20114229	1.109447504	3.151866127
C	-5.709817526	1.928755492	5.097611106
H	-6.426887229	1.722642663	5.922799862
H	-4.675456271	1.833542882	5.495911459
C	-5.925520353	3.356970059	4.583475135
H	-6.959936628	3.452465795	4.185314551
H	-5.20847663	3.563255943	3.758267884
C	-5.717157204	4.382152312	5.70392524
H	-6.433387363	4.184461049	6.531145106
H	-4.683594145	4.295040011	6.10457511
C	-5.930895352	5.805059451	5.195976129
H	-6.966411518	5.926467353	4.812499504
H	-5.773655449	6.524077547	6.027394743
H	-5.209123401	6.037512255	4.384125951
C	17.04978102	-5.329714835	4.559527049
H	16.06965017	-5.741600547	4.888210217
H	17.79553631	-5.580625191	5.346417973
C	17.46255564	-6.032447053	3.251310994
H	16.71742033	-5.814256533	2.455944734
H	18.4539619	-5.655581008	2.918806921
C	17.54852148	-7.550296509	3.447479538
H	18.29766753	-7.777159167	4.237932204
H	16.55826867	-7.935137069	3.777365943
C	17.95650633	-8.250760085	2.146047745
H	17.20782439	-8.022778857	1.355380245
H	18.94700853	-7.866270494	1.816338313
C	18.04132173	-9.769093121	2.341038111

H	18.79038001	-9.997134575	3.131300302
H	17.05088271	-10.15339666	2.67112513
C	18.448511	-10.46940055	1.039336779
H	17.69953555	-10.24140797	0.248939705
H	19.43910344	-10.08546825	0.709203058
C	18.53287057	-11.98747422	1.234699935
H	17.54481232	-12.37907756	1.561426002
H	19.28274622	-12.22334102	2.02109238
C	18.93779094	-12.68813347	-0.058929689
H	18.99060919	-13.78391301	0.112668585
H	18.19001435	-12.48911072	-0.855992719
H	19.93539448	-12.33269061	-0.394390983
C	16.36904419	-1.685289568	5.826135885
H	15.60020519	-1.374295366	5.086123093
H	17.33565772	-1.21037792	5.551224687
C	15.94984324	-1.190077567	7.214942241
H	16.71789086	-1.492685105	7.960621876
H	14.98049449	-1.658381807	7.495252672
C	15.79950478	0.33567763	7.232503302
H	15.03194714	0.63820065	6.486189222
H	16.76891366	0.803997765	6.952215715
C	15.379537	0.83173995	8.621066691
H	14.41022755	0.363238895	8.901348985
H	16.14725162	0.529511501	9.367310256
C	15.22883486	2.357407128	8.638288278
H	14.46109513	2.659815037	7.892101281
H	16.19813183	2.826134777	8.358245043
C	14.80876884	2.852899306	10.02676513
H	13.83832915	2.391139908	10.31204836
H	15.57368532	2.557397087	10.77772524
C	14.6576345	4.371147218	10.04788418
H	13.87933834	4.691617241	9.322872051
H	14.35348523	4.70030804	11.06381465
H	15.62212683	4.858598446	9.790524923
C	5.280031804	-7.35740147	-2.420604227
H	4.298134455	-7.055050747	-1.995578087
H	5.316731481	-6.99276349	-3.470090657
C	5.384810378	-8.881778394	-2.438257086

H	6.38359248	-9.198640814	-2.806743935
H	5.214450794	-9.298550203	-1.42397166
C	4.369144805	2.897789206	-0.878336758
H	5.105607798	2.262411909	-0.339436624
H	4.92518152	3.598502023	-1.538720865
C	3.576425734	3.706734843	0.144250385
H	2.849814885	4.371921671	-0.36919502
H	3.029654141	3.027960647	0.833030487
C	1.412272902	0.265636547	-5.257307511
H	1.714242804	-0.664343639	-5.779607737
H	0.712170487	0.0133224	-4.432492444
H	-18.92649051	-0.866087764	1.467266117
H	0.87642734	0.908621848	-5.986967453
H	4.271365724	4.333958077	0.741402986
H	9.578921554	-8.329685863	2.165115806
H	4.609802302	-9.296711651	-3.116547838
C	-19.60569978	1.151400668	1.518823121
H	-20.04503269	1.137559293	2.538913391
H	-18.55055492	1.489439037	1.593510828
H	-20.15252433	1.881340534	0.886867342
H	-19.29042854	-0.197727319	-0.151560893
H	-23.82855152	2.11224349	-3.468611581
H	-16.68573548	-8.434985199	5.09319727
C	-17.94999692	-7.64001642	3.56972807
H	-18.49041463	-6.715515457	3.293132767
H	-17.29124254	-7.94289413	2.728314573
H	-18.70063098	-8.441752441	3.732942488
H	-14.38429493	-7.761128556	5.151577936
C	-14.9675615	-6.814732679	7.02252554
H	-15.80164029	-7.508376365	7.262681144
H	-15.23115759	-5.801223689	7.393276654
C	-13.70099086	-7.288634145	7.744691533
H	-13.42878172	-8.308290902	7.394664166
C	-13.9084589	-7.325893854	9.256034718
H	-14.73063825	-8.026445672	9.515511478
H	-12.97687325	-7.67299947	9.750619952
H	-14.15608959	-6.312080889	9.636742949
H	-12.85834427	-6.600617812	7.514383947

2DTFDPP20

C	-2.746167709	3.275749105	7.133286142
C	-3.752134197	3.963697758	6.461258987
C	-3.45812472	5.071035693	5.616899655
C	-2.156040505	5.507867593	5.458627907
C	-1.115291368	4.796615116	6.088202489
C	-1.430502431	3.692371523	6.921845299
H	-2.970380859	2.424241226	7.769360511
H	-1.915781721	6.354007989	4.818895911
C	-4.747567484	5.565631315	4.936077092
C	-5.191151877	3.701328702	6.421052836
C	-5.806541603	4.625990743	5.531425289
C	-5.934651502	2.729482011	7.087447445
C	-7.31010878	2.702803229	6.859562725
H	-5.464168132	2.026495658	7.76872082
C	-7.944030417	3.613900286	5.97327295
C	-7.167494372	4.57614079	5.298100932
H	-7.653467079	5.271417405	4.61767789
S	-8.483147565	1.626230846	7.602995643
C	-9.813422114	2.416614567	6.737406582
C	-9.357557835	3.422567248	5.924603171
H	-10.01403708	4.032606987	5.312393319
S	0.027249071	2.938225546	7.549820563
C	1.032708336	4.07444754	6.665973236
C	0.303636219	4.983813865	5.960356191
H	0.7317835	5.766710112	5.34268799
C	-11.17839679	2.02391309	6.983522451
C	-11.67404732	0.974961175	7.73777441
S	-12.49223679	2.973636307	6.330602548
C	-13.08094286	0.944169645	7.818341183
H	-11.03379878	0.250892047	8.229944121
C	-13.69889194	1.9864934	7.143459947
H	-13.64391725	0.185822176	8.347795021
C	-15.12230158	2.21901639	7.100860831
C	-16.10421243	1.248626547	7.241530703
N	-15.80115969	3.443008656	6.930493321
C	-17.39143839	1.838099688	7.132654557

C	-17.22938673	3.266987449	6.918218197
O	-18.03398455	4.173782685	6.744034659
C	-16.256219	-0.1730136	7.483797418
N	-17.68684647	-0.35448543	7.479842703
C	-18.37099568	0.863572431	7.295216185
O	-15.44431747	-1.067088717	7.678761764
C	-19.79778542	1.108905076	7.319768984
C	-20.40506571	2.249129641	6.815075614
S	-21.01222657	0.071817495	8.068161652
C	-21.80883545	2.273597767	7.002208487
H	-19.83731646	3.048308974	6.354086808
C	-22.28168729	1.167351361	7.658343815
H	-22.44455091	3.085046547	6.666138927
H	-23.30011205	0.933944074	7.939032771
C	21.21946565	0.624802967	5.118032059
C	20.63204968	1.331161876	6.163748763
C	21.28077636	1.473315523	7.421376555
C	22.51982941	0.895008034	7.639959033
C	23.13554326	0.172979689	6.599330604
C	22.47241304	0.054053094	5.349056717
H	20.72469375	0.521946381	4.156445185
H	23.02573967	0.98421873	8.598644622
C	20.40452822	2.305052282	8.366495631
C	19.35143879	2.034705806	6.21251435
C	19.16523387	2.573750939	7.518967426
C	18.39400105	2.195516955	5.215036873
C	17.22143604	2.87077564	5.552727249
H	18.54284755	1.790743314	4.21830954
C	16.99463477	3.379183663	6.860137557
C	17.99538197	3.235983419	7.843840901
H	17.83136322	3.642026194	8.839085392
S	15.82919223	3.140273832	4.515789428
C	14.9387763	3.89138419	5.84954181
C	15.69274778	3.953373487	6.99235372
H	15.32469466	4.386511136	7.916790795
S	23.44390723	-0.865746977	4.207866754
C	24.70496222	-1.074541727	5.405253583
C	24.41308438	-0.489226731	6.595144959

H	25.07796964	-0.510253687	7.452114853
C	13.54665098	4.244193583	5.689193453
C	12.8357922	4.6183019	4.563399565
S	12.4815602	4.093290981	7.058308668
C	11.45834941	4.799558818	4.797582587
H	13.30249553	4.762488838	3.595060357
C	11.07510237	4.569487587	6.115646814
H	10.74263696	5.054548133	4.025226231
C	9.706858353	4.575262889	6.58182132
C	8.589509212	4.502438288	5.755156171
N	9.228076305	4.532006228	7.914479604
C	7.413916893	4.38037764	6.553881678
C	7.784968212	4.450764455	7.959684804
O	7.11832063	4.46845269	8.977216451
C	8.200021933	4.41866075	4.356013694
N	6.748473149	4.332841699	4.404619325
C	6.329459625	4.238117188	5.738487994
O	8.845759384	4.441701237	3.315558585
C	4.935466159	4.034789802	6.187018454
C	4.59540288	3.206513128	7.239897061
S	3.51823093	4.873309993	5.453080994
C	3.242110104	3.16287869	7.487619071
H	5.314054193	2.624345493	7.802063866
C	2.505032795	3.974637591	6.644762995
H	2.810749053	2.546875088	8.265548599
H	25.59828291	-1.624942357	5.140535052
C	-5.019196118	7.052998709	5.273109837
C	-4.680226127	5.395566564	3.394199
H	-5.685775581	5.614106401	2.977690638
H	-4.037635319	6.193722679	2.961862261
C	-4.180265131	3.986541072	2.920748597
H	-4.213683043	3.284117985	3.779910994
C	-5.097365168	3.368344746	1.835984851
H	-4.597861757	2.466406792	1.417902739
H	-5.242890189	4.091781075	1.00379034
C	-6.463849023	2.94312653	2.401578592
C	-2.709488655	4.053895519	2.414119868
H	-6.316449723	2.341748438	3.325897505

H	-7.055901991	3.845175309	2.654836588
C	-7.265723909	2.113329224	1.394748463
H	-7.41774889	2.706627215	0.466204355
H	-6.697682791	1.19167665	1.139633631
C	-8.628591275	1.724942579	1.983419164
H	-8.473207494	1.138053122	2.916010318
H	-9.195503783	2.647387765	2.24018745
C	-9.443999908	0.890371653	0.99053888
H	-9.608958917	1.472755181	0.057868612
H	-8.886816506	-0.036885509	0.733644419
C	-10.79852306	0.505920242	1.579410495
H	-10.66116617	-0.098627357	2.501327484
H	-11.38620961	1.416734485	1.822945498
H	-11.36753822	-0.097005138	0.840765015
H	-2.198354504	4.957834458	2.804005696
H	-2.682421967	4.140992973	1.305208111
H	-4.141437704	7.63463461	4.914241086
H	-5.869065947	7.430072272	4.662731357
C	-5.271922168	7.348369526	6.790156541
H	-4.790673213	6.542727804	7.386137681
C	-6.798884971	7.397210136	7.116239063
C	-4.562519756	8.662332838	7.202281337
H	-7.1277654	8.437104601	7.331772191
H	-3.498280292	8.609639046	6.880935103
H	-5.033075874	9.524409275	6.679788147
H	-7.402170368	7.08350929	6.240884387
C	-4.581790087	8.899953951	8.721139648
H	-5.625857993	9.061096449	9.063723361
H	-4.171352459	8.0060716	9.240512733
C	-3.745559113	10.12914631	9.09635921
H	-2.693700313	9.973140788	8.769491089
H	-4.150241853	11.02276682	8.571687859
C	-3.773718695	10.37286643	10.60983258
H	-4.824935586	10.53134958	10.93771349
H	-3.370870703	9.478141997	11.13411682
C	-2.93548309	11.60021543	10.98486203
H	-1.882042641	11.44740134	10.66349631
H	-3.334776111	12.49954258	10.4670429

C	-2.961025118	11.8459802	12.49061113
H	-2.346836313	12.73900985	12.73193965
H	-4.001709611	12.02718828	12.83445475
H	-2.542790227	10.97051368	13.03166261
C	-7.17539885	6.494680334	8.303240712
H	-6.704087939	6.881814041	9.231683258
H	-6.791544663	5.468424759	8.126060333
C	-8.696769282	6.430349035	8.489815718
H	-9.085466189	7.440990476	8.74133973
H	-9.17960967	6.092767295	7.5467864
C	-9.075795038	5.457753609	9.602495812
H	-8.612403052	5.769947293	10.56243237
H	-10.17893827	5.445834063	9.727546628
H	-8.737503531	4.430743199	9.347229457
C	-1.886540632	2.834254094	2.857097838
H	-1.932019042	2.744702613	3.964778583
H	-2.317290435	1.911713312	2.410702728
C	-0.421154946	2.97670055	2.42968284
H	-0.360770483	2.968426347	1.32024469
H	-0.008499914	3.940801474	2.800492011
C	0.432705012	1.842092322	2.986446704
H	0.414382963	1.856491396	4.097508102
H	0.056240623	0.862023722	2.623962816
H	1.483177523	1.968317405	2.649099321
C	-18.26702233	-1.681273386	7.4454624
C	-15.20544849	4.763917802	6.940954283
H	-17.50593525	-2.441934069	7.715397048
H	-19.0422026	-1.761318768	8.2321136
H	-14.46512313	4.821803886	6.122568308
H	-15.96392285	5.537768933	6.691877169
C	-18.86489697	-2.014475578	6.053759346
H	-19.57584708	-1.195780676	5.810741333
C	-19.73165985	-3.305138892	6.04569238
C	-17.82205168	-1.966247652	4.905687033
H	-20.74676669	-3.044248906	6.420055934
H	-19.86312105	-3.650411417	4.996523564
H	-18.35702134	-2.009852513	3.931777748
H	-17.29621374	-0.987757687	4.925407376

C	-19.20346803	-4.465392346	6.912385074
H	-19.29011641	-4.190803075	7.986064987
H	-18.13616964	-4.666663577	6.694442833
C	-20.0124341	-5.744505025	6.667543971
H	-21.08552897	-5.558290277	6.891573525
H	-19.92213169	-6.046461777	5.601256321
C	-19.51566297	-6.888946717	7.546320306
H	-19.61911917	-6.624014074	8.620179014
H	-20.11801776	-7.799944929	7.345850584
H	-18.4502118	-7.111558791	7.324098147
C	-16.77658901	-3.081168563	4.92335574
H	-16.00906261	-2.872123184	4.148706727
H	-16.26454346	-3.141347937	5.904391633
H	-17.24490774	-4.055821688	4.675528283
C	6.012950115	3.785937584	3.282310941
C	9.949136234	4.960891099	9.095969464
H	6.683982679	3.1886944	2.625947474
H	5.259015955	3.063770932	3.664851558
H	10.95355745	5.34631846	8.838791736
H	9.409542667	5.827920476	9.530385685
C	5.312534856	4.891980226	2.44955761
C	10.05971148	3.824837381	10.14972611
H	4.754643359	5.547135404	3.148189284
H	9.033090395	3.479622987	10.40059187
C	4.280852056	4.244628178	1.489242751
C	6.329666118	5.762803039	1.660070598
C	10.84880852	2.616576835	9.582006721
C	10.73004705	4.352115632	11.44532372
H	7.237038257	5.176756046	1.403225757
H	5.897958793	6.093587515	0.691396905
C	6.731455355	7.028009794	2.420128328
H	7.487897156	7.589465302	1.832425915
H	5.844969248	7.680828731	2.568076104
H	7.165643929	6.784378691	3.409390092
H	4.796424558	3.87287761	0.575842747
H	3.801330471	3.36596536	1.974566153
C	3.154015154	5.217572899	1.106355396
H	3.577049841	6.149043803	0.673553036

H	2.584534415	5.486244082	2.02390748
C	2.202854435	4.579723936	0.087532161
H	2.745531623	4.400131754	-0.865999137
H	1.840160386	3.602102235	0.470218158
C	1.000933338	5.479238995	-0.186100678
H	1.335829214	6.456520796	-0.594291404
H	0.333979559	4.992392899	-0.928396583
H	0.424711116	5.649562358	0.748418426
H	10.56341971	2.447378788	8.521974275
H	11.9389366	2.834857265	9.611196887
C	10.57114918	1.304563142	10.33295584
H	10.86324114	1.399202251	11.3990912
H	9.483494202	1.07698815	10.28461089
C	11.35904496	0.146312231	9.707262958
H	11.06618499	0.023689509	8.641571927
H	12.44758967	0.369141093	9.748123078
C	11.09685476	-1.163524278	10.44398916
H	11.67981332	-1.980094256	9.968145691
H	11.40855436	-1.077561085	11.50675368
H	10.01772699	-1.42293555	10.39731169
H	10.99178533	3.506729061	12.11330288
H	11.67826805	4.878377937	11.19901624
C	9.826038894	5.289209068	12.24929907
H	8.836598522	4.817853713	12.42933511
H	10.29723239	5.500858881	13.2323805
H	9.687137153	6.257169468	11.72669524
C	20.08054974	1.520765614	9.678673327
H	21.05400769	1.29324696	10.1692656
H	19.5682825	2.208415916	10.3871312
C	21.12763775	3.630555604	8.792902556
H	22.0704416	3.356318626	9.312028016
H	20.51717394	4.113032605	9.588263056
C	19.25949484	0.184468743	9.669492958
H	19.45423712	-0.205785545	10.69605864
C	21.45701085	4.770077389	7.76179675
H	22.0182209	5.526534064	8.356399051
C	19.81670388	-0.914408058	8.733488271
H	19.48652207	-0.739505336	7.68896967

H	20.92404983	-0.87513033	8.7539046
C	17.70821148	0.376708305	9.643666366
H	17.26587985	-0.270101989	10.43444496
H	17.45703528	1.416375643	9.941523838
C	20.18417234	5.490378403	7.245812428
H	19.76680041	4.943614858	6.373364463
H	19.42069396	5.50648824	8.052345228
C	22.3915854	4.368915226	6.592560829
H	21.93858338	3.561601281	5.993429226
H	22.49989429	5.224074568	5.89475053
C	19.43306853	-2.333137346	9.188267193
H	18.33900735	-2.414621994	9.34983451
H	19.93365465	-2.549623556	10.15784217
C	19.87071259	-3.373822246	8.150813207
H	19.36225918	-3.163436891	7.183868311
H	20.96988899	-3.298997895	7.995272568
C	19.51794157	-4.793187367	8.609680355
H	18.41870416	-4.867376262	8.764155732
H	20.02631078	-5.004467018	9.576383203
C	19.95489327	-5.830928507	7.56957136
H	19.44784183	-5.627724413	6.601067754
H	21.05368482	-5.764805674	7.412449555
C	19.60548687	-7.245676211	8.021917996
H	19.93307064	-7.974092805	7.250512488
H	20.12074654	-7.483805613	8.976845385
H	18.50802876	-7.346060911	8.161918164
C	16.97805068	0.03241444	8.329661545
H	17.45294463	0.538724469	7.473033712
H	17.02728749	-1.058363301	8.1381139
C	15.50026604	0.441413898	8.407926158
H	15.00632011	-0.102972369	9.241990604
H	15.41880838	1.531005281	8.608908822
C	14.76373891	0.125703464	7.109975635
H	14.776775	-0.967878475	6.916540542
H	13.70822738	0.461765173	7.190710859
H	15.23932336	0.653295513	6.257271708
C	23.81126931	3.994311706	7.046356086
H	24.31351324	4.894496239	7.463471352

H	23.77586737	3.230722746	7.844982069
C	24.63371228	3.452531741	5.869977388
H	24.79604918	4.265819992	5.128928248
H	24.07118922	2.633745186	5.370790778
C	25.98706372	2.910648197	6.342564804
H	26.57526692	3.736035924	6.800672989
H	25.82047137	2.126119465	7.113268075
C	26.77436888	2.308661993	5.173359072
H	26.98605196	3.097902033	4.419464912
H	26.17397549	1.509414867	4.686482806
C	28.0938025	1.708625258	5.648811179
H	28.6447432	1.288154556	4.781314682
H	28.72344547	2.489670596	6.125788735
H	27.90463362	0.89223551	6.378309593
C	20.43412344	6.952650685	6.839378923
H	20.85487258	7.510173457	7.704931842
H	21.16068864	7.001453769	6.00219394
C	19.12785263	7.623675701	6.396469533
H	18.69666963	7.071154471	5.53302956
H	18.39208464	7.601346594	7.229916736
C	19.36327251	9.074351124	5.986344314
H	19.77220283	9.655190043	6.840418048
H	20.07442909	9.124010857	5.134379178
H	18.4016049	9.532664329	5.673133761
C	-14.57013074	5.114333857	8.316242545
H	-13.82971603	4.332538924	8.57652983
C	-13.79932615	6.457999779	8.239238005
H	-14.4543959	7.252613484	7.819958663
C	-15.65253719	5.191384602	9.437078424
H	-16.63271946	5.474569308	8.995261973
H	-15.42136269	5.995300617	10.16981555
C	-15.83179638	3.881685911	10.22724655
H	-16.78182548	3.952927481	10.80099616
H	-15.93193304	3.020765151	9.540848166
C	-14.68447529	3.627342591	11.21673523
H	-13.71198596	3.572291623	10.68509615
H	-14.63318629	4.460076142	11.95135784
C	-14.89306182	2.316415924	11.96916909

H	-14.91711274	1.463828631	11.25683651
H	-14.0564451	2.159480057	12.68217614
H	-15.84576083	2.345468004	12.53969335
C	-12.5082173	6.375873025	7.420625996
H	-11.86729151	5.546296345	7.785811558
H	-12.72337048	6.234196567	6.342501692
H	-11.94172079	7.324645275	7.530654614
H	-13.50300843	6.772761248	9.263947653

2DTFDPP32

C	3.761169598	3.620777649	-0.000261759
C	4.241657509	2.409222611	-0.497502957
C	3.388965895	1.411275873	-1.06243671
C	2.036710838	1.694535618	-1.169307341
C	1.524784704	2.906723168	-0.67003107
C	2.391006866	3.855123792	-0.074526859
H	4.436209825	4.348802614	0.439843173
H	1.300841922	1.022188969	-1.589989339
C	4.223347116	0.160270802	-1.456854292
C	5.62150014	1.94533885	-0.523773043
C	5.655821429	0.650412129	-1.094207273
C	6.776960507	2.572539804	-0.058452319
C	7.97555981	1.87156074	-0.165884962
H	6.741924047	3.559923069	0.392362268
C	8.038215656	0.572787262	-0.739733871
C	6.854675566	-0.033042363	-1.209038688
H	6.90118322	-1.036848292	-1.625246963
S	9.555922453	2.39275193	0.396933661
C	10.28604384	0.860703915	-0.119033408
C	9.35975413	0.033691278	-0.70257727
H	9.603994101	-0.955598402	-1.076230723
S	1.490226932	5.241995383	0.524177953
C	-0.041065925	4.52876957	-0.024106411
C	0.160677527	3.31910627	-0.63642979
H	-0.646827771	2.718967366	-1.043546774
C	11.67845532	0.598496914	0.140770072
C	12.61715725	1.406165782	0.762125065
S	12.39775125	-0.928896437	-0.316780683

C	13.88558762	0.811345079	0.894466104
H	12.38788932	2.403865287	1.120480134
C	13.94521546	-0.480965149	0.38958335
H	14.74871699	1.298371928	1.33300158
C	15.1010261	-1.335741811	0.417860797
C	16.42347497	-0.92820283	0.53811517
N	15.1345236	-2.735483768	0.20690182
C	17.29453119	-2.053655066	0.419426961
C	16.48813529	-3.255953847	0.238507659
O	16.78533395	-4.430428818	0.1094671
C	17.22798308	0.265625446	0.7538599
N	18.57511987	-0.254777453	0.788771655
C	18.61173715	-1.653740077	0.570867922
O	16.9404491	1.442572199	0.921843557
C	19.79586351	-2.475508589	0.680949794
C	20.94557925	-2.246574041	1.422148812
S	19.89650121	-4.030657589	-0.128347948
C	21.90207675	-3.28550404	1.313259347
H	21.07572536	-1.37934872	2.060207947
C	21.48000644	-4.304389439	0.49672158
H	22.85668519	-3.282883477	1.827342497
H	22.00610086	-5.215061122	0.241940886
C	-16.36371158	-5.080371444	-1.607392524
C	-16.05579233	-4.418259956	-0.422407379
C	-16.56457499	-4.867303363	0.830248717
C	-17.30455004	-6.032592221	0.909103136
C	-17.64170324	-6.713435448	-0.277457543
C	-17.18380637	-6.207638975	-1.522566417
H	-16.00535336	-4.717977911	-2.566596533
H	-17.68163436	-6.39715937	1.862061893
C	-16.20595615	-3.854472386	1.930240836
C	-15.27904445	-3.194461781	-0.213114217
C	-15.38592665	-2.81556841	1.153513673
C	-14.52695077	-2.443328164	-1.11596888
C	-13.88495601	-1.304475206	-0.627655147
H	-14.42051027	-2.748253616	-2.152958377
C	-14.03268776	-0.877227695	0.718564801
C	-14.81754995	-1.640975396	1.604122359

H	-14.91885347	-1.318664427	2.637506583
S	-12.79338905	-0.238174194	-1.502675172
C	-12.51541669	0.739354075	-0.049519097
C	-13.25669601	0.282692136	1.010551191
H	-13.19587255	0.716455911	2.002412073
S	-17.81857566	-7.159391606	-2.858174
C	-18.65396412	-8.220221298	-1.743479573
C	-18.4736459	-7.875585891	-0.442358176
H	-18.91962084	-8.41323433	0.387589483
C	-11.50289518	1.76616135	0.008872727
C	-11.23652888	2.647714234	1.046555368
S	-10.26865337	1.891080237	-1.212771836
C	-10.02025062	3.337578211	0.910263759
H	-11.88747094	2.755918678	1.906502112
C	-9.308413909	3.005881466	-0.241217653
H	-9.635596107	4.043940932	1.637625046
C	-7.949222663	3.414450705	-0.501017199
C	-7.258125091	4.411447527	0.18963475
N	-7.01887586	2.864141199	-1.408404359
C	-5.910548165	4.471257517	-0.253249008
C	-5.728258158	3.488019043	-1.310923793
O	-4.764335469	3.175638396	-1.993859176
C	-7.448219187	5.408783134	1.225129917
N	-6.167027867	6.048073493	1.305230551
C	-5.213795177	5.449366481	0.453254985
O	-8.410430146	5.70305357	1.927369399
C	-3.780338594	5.663634722	0.441978953
C	-2.953500844	6.691418418	0.890301596
S	-2.782964525	4.34072571	-0.158887432
C	-1.576610573	6.420706814	0.767152956
H	-3.285710318	7.640613325	1.289513971
C	-1.306104937	5.174928455	0.227303386
H	-0.805194917	7.124106281	1.06181552
H	-19.23747098	-9.042876123	-2.135497442
C	3.962981155	-1.122193503	-0.603559298
C	4.216080264	-0.11312141	-2.988133333
H	5.025409511	-0.835589452	-3.2346645
H	4.546449793	0.825702236	-3.484804234

C	2.881798379	-0.609117346	-3.631419045
H	2.048199989	-0.439693467	-2.941756369
C	2.560436255	0.182721495	-4.927116692
H	1.79123149	-0.352046463	-5.522967922
H	3.47205486	0.253336922	-5.561045165
C	2.018772018	1.589793876	-4.623012887
C	2.930210353	-2.132845473	-3.923288798
H	1.075083716	1.497417946	-4.042014257
H	2.751379613	2.159042463	-4.012571585
C	1.734582971	2.369300282	-5.910882724
H	2.676313628	2.472106678	-6.493910658
H	0.995895212	1.809082829	-6.525658659
C	1.178128877	3.762465333	-5.592571268
H	0.238956713	3.658409086	-5.005090084
H	1.917638594	4.322810493	-4.978523955
C	0.887820802	4.546635163	-6.876868927
H	1.823532542	4.659838842	-7.466732582
H	0.146579171	3.993859447	-7.494386417
C	0.332025473	5.932387005	-6.561839638
H	-0.618418965	5.846857742	-5.993060382
H	1.065393962	6.515851725	-5.965303231
H	0.131429364	6.47653034	-7.508730804
H	3.4515082	-2.316972423	-4.888712296
H	3.514859349	-2.653045091	-3.13826508
H	4.589971293	-1.098106646	0.317662052
H	4.381787112	-1.986041337	-1.15972739
C	2.502554027	-1.417232595	-0.136594425
H	1.802588793	-1.07954756	-0.915464622
C	2.200840589	-0.675161786	1.202123538
C	2.311509301	-2.951480657	-0.004542324
H	2.493638923	-1.304603968	2.070737428
H	2.664751743	-3.426838042	-0.945509727
H	2.943637598	-3.347812733	0.820375263
H	2.833127735	0.233714314	1.262970981
C	0.850781592	-3.37598923	0.216161714
H	0.586344173	-3.23300982	1.284445863
H	0.177074598	-2.753938792	-0.413594785
C	0.643019237	-4.854079412	-0.137968227

H	0.960684125	-5.031037695	-1.18747534
H	1.270481326	-5.485048706	0.529408038
C	-0.827832685	-5.254618639	0.013082826
H	-1.125519587	-5.142813869	1.07678784
H	-1.460865796	-4.581597522	-0.606893242
C	-1.052415078	-6.705004889	-0.426994903
H	-0.743610573	-6.827085064	-1.488301489
H	-0.437132197	-7.386518956	0.200020259
C	-2.520368889	-7.099330236	-0.294199231
H	-2.654692786	-8.14951979	-0.62867267
H	-2.847569555	-7.01979892	0.763774858
H	-3.15557034	-6.44148396	-0.925012494
C	0.731585512	-0.234205936	1.361094126
H	0.112396654	-1.091032624	1.690744119
H	0.32493112	0.12704867	0.394184718
C	0.603411034	0.887661095	2.400828148
H	0.969237496	0.526610113	3.386622324
H	1.22206649	1.759351673	2.09468388
C	-0.842876526	1.349181413	2.544303906
H	-1.479425473	0.510583921	2.895924697
H	-0.898974704	2.173088498	3.286270667
H	-1.226523042	1.723360702	1.571454293
C	1.528461362	-2.767536906	-3.954941643
H	1.017001426	-2.5782646	-2.985519347
H	0.921290171	-2.311073699	-4.764584962
C	1.610837639	-4.280419786	-4.185159076
H	2.125708487	-4.485786419	-5.148868833
H	2.196489375	-4.752915201	-3.366936966
C	0.219521403	-4.907515873	-4.224775368
H	-0.327823818	-4.702212997	-3.280406177
H	-0.362042251	-4.498361618	-5.078106194
H	0.310294696	-6.006913255	-4.350619966
C	19.62805033	0.620251697	0.287151851
C	14.07984421	-3.612238025	0.707943059
H	19.6744541	1.508667709	0.95444631
H	20.62170867	0.144384795	0.374831758
H	13.20674802	-3.517107089	0.036387819
H	14.38136293	-4.679116635	0.618402363

C	19.41088431	1.10832748	-1.176763946
H	18.44269358	1.653327069	-1.20744975
C	20.50548961	2.143562809	-1.540704217
H	20.50072446	2.942524056	-0.766192731
H	21.51022665	1.669403115	-1.511606556
C	20.28433534	2.807052303	-2.909962163
H	20.42258579	2.055510523	-3.715966343
H	19.24755656	3.205677554	-2.966374469
C	-5.952281121	7.19652805	2.154302872
C	-7.214200695	1.696196904	-2.239950365
H	-6.723153131	7.242011758	2.954754239
H	-4.991256706	7.070773741	2.693633485
H	-8.120736928	1.862345836	-2.855222176
H	-6.398665278	1.598559632	-2.988812162
C	-5.985274997	8.517500732	1.340399962
C	-7.304413233	0.390264736	-1.396172666
H	-5.263986217	8.424367264	0.499592102
H	-7.895804539	0.606808768	-0.484316072
C	-5.512020027	9.688326335	2.236673354
C	-7.404507953	8.788989515	0.760384897
C	-8.057372696	-0.708073299	-2.192116844
C	-5.900458812	-0.106203816	-0.952112734
H	-8.177859014	8.304272831	1.393829195
H	-7.637546854	9.873576345	0.784712598
C	-7.537728413	8.317575833	-0.700832749
H	-7.056681864	7.326893444	-0.828298545
H	-6.3342361	9.994833636	2.920838442
H	-4.660038918	9.355143669	2.869837097
C	-5.029929621	10.89511305	1.414175564
H	-5.846144892	11.26638426	0.759347028
H	-4.181281553	10.57991958	0.767688562
C	-4.567659979	12.03404874	2.330753081
H	-5.415954595	12.35347455	2.975646005
H	-3.74437459	11.66970258	2.984265863
C	-4.071952488	13.23312489	1.513856073
H	-4.895468163	13.60028576	0.862249107
H	-3.225335516	12.91273578	0.867177756
H	-8.81462424	-0.244863959	-2.861152332

H	-7.349429495	-1.26322459	-2.84659641
C	-8.80448308	-1.687925368	-1.269714496
H	-8.077516922	-2.254515906	-0.648839154
H	-9.477841928	-1.117908665	-0.593097798
C	-9.649796485	-2.663803278	-2.09501352
H	-10.33776552	-2.088125619	-2.753392597
H	-8.975983627	-3.272237448	-2.73788147
C	-10.48266449	-3.593017235	-1.203954345
H	-9.803797957	-4.183334981	-0.549968155
H	-11.15918527	-2.988156186	-0.561206069
H	-5.902002046	-1.213199407	-0.853010855
H	-5.146896995	0.136403023	-1.732665186
C	-5.472425809	0.471987898	0.410568612
H	-5.805863232	1.52280707	0.506826632
C	-17.47809783	-3.199153765	2.539340301
H	-17.14782964	-2.435398811	3.273252817
H	-18.02612274	-3.947248686	3.153522748
C	-15.36379586	-4.485237821	3.072768371
H	-16.04158074	-5.111252157	3.695055628
H	-15.03971839	-3.677333988	3.762543897
C	-18.45461309	-2.56131929	1.492048927
H	-17.89451815	-2.358176237	0.560034949
C	-14.12997606	-5.34228543	2.613321759
H	-14.02721231	-5.289763201	1.50935537
C	-19.03814417	-1.20800391	1.980212282
H	-19.88546855	-0.908270113	1.32441361
H	-19.45783352	-1.346530522	3.000915564
C	-19.62210141	-3.532359549	1.144421287
H	-19.37847007	-4.57179754	1.434804253
H	-20.53527184	-3.272513616	1.724816605
C	-14.37377215	-6.832303147	2.971976319
H	-14.4802395	-6.942189576	4.073932657
H	-15.33627025	-7.143111286	2.513097888
C	-12.79004767	-4.837035986	3.220558752
H	-12.85050607	-4.839209172	4.330959992
H	-11.9706433	-5.52406544	2.925041659
C	-18.03530441	-0.037718035	2.018529968
H	-17.14964101	-0.29688861	2.630966044

H	-18.53733089	0.814545911	2.526922147
C	-17.58869538	0.426166946	0.622679987
H	-17.00762249	-0.376350343	0.124279522
H	-18.48173095	0.653020127	0.000183523
C	-16.70919078	1.678892549	0.720736103
H	-15.86405228	1.482679889	1.415894361
H	-17.31089343	2.51961665	1.130901477
C	-16.15330445	2.073289171	-0.652287798
H	-15.56915951	1.229472187	-1.07829332
H	-16.9926236	2.302747269	-1.344199123
C	-15.24582462	3.29573294	-0.549152145
H	-14.83892091	3.542433678	-1.552320528
H	-15.81848495	4.169388784	-0.17167652
H	-14.39664544	3.09167366	0.136886974
C	-19.95209683	-3.521394016	-0.355982568
H	-20.30318107	-2.508133469	-0.649838651
H	-19.0331112	-3.758073974	-0.935549762
C	-21.02904422	-4.558238993	-0.694100869
H	-20.69665065	-5.567343106	-0.365494943
H	-21.97158617	-4.305937943	-0.16138333
C	-21.29950256	-4.600449102	-2.1948348
H	-20.37682987	-4.885767039	-2.744322697
H	-22.08660103	-5.353568887	-2.409505021
H	-21.64855895	-3.608303866	-2.552208467
C	-12.4035894	-3.436176913	2.725131553
H	-12.58081835	-3.365731393	1.630105364
H	-13.02752768	-2.689291889	3.254711344
C	-10.93219653	-3.104141942	2.995483794
H	-10.28420377	-3.792206825	2.408800324
H	-10.71452009	-3.242330914	4.077425719
C	-10.63167863	-1.653340525	2.595461571
H	-10.86980135	-1.508520351	1.518965523
H	-11.27747529	-0.970780786	3.191298266
C	-9.161706493	-1.296789762	2.836602083
H	-8.51402246	-1.91311759	2.175315668
H	-8.890536014	-1.506638129	3.894129324
C	-8.906695142	0.179702828	2.548763442
H	-7.837274812	0.417398834	2.723809058

H	-9.159217185	0.412186324	1.494963242
H	-9.523590592	0.816789758	3.217641745
C	-13.26663071	-7.782807301	2.484296232
H	-12.90115321	-7.457900299	1.489589887
H	-12.4139834	-7.747562213	3.196725766
C	-13.77320661	-9.227223062	2.395705
H	-14.15814661	-9.549821572	3.387645271
H	-14.60370984	-9.287869959	1.659570773
C	-12.66157179	-10.17878586	1.96338903
H	-12.26678342	-9.887559925	0.96741763
H	-11.83338284	-10.16572425	2.703504628
H	-13.0625614	-11.2120151	1.896821899
C	13.66369223	-3.3308458	2.184242132
H	13.37107205	-2.267735881	2.275713314
C	12.44543308	-4.215775107	2.572708742
H	12.61557011	-5.251054773	2.203405777
C	14.84433098	-3.579554934	3.159138227
H	14.9793487	-4.671735619	3.321595987
H	15.78497099	-3.191813503	2.724140611
C	14.65844305	-2.862887304	4.507873733
H	14.51086624	-1.77509842	4.327256359
H	13.76370583	-3.253836167	5.034773185
C	15.88622667	-3.058132395	5.405923621
H	16.03888281	-4.144920984	5.587799709
H	16.78706123	-2.655765435	4.891569517
C	15.70771041	-2.338567289	6.747883556
H	15.55143495	-1.252437727	6.564772579
H	14.80978245	-2.742745745	7.265504506
C	-11.32069817	-4.540750039	-2.069808834
H	-10.64499171	-5.09928353	-2.754975821
H	-12.03029882	-3.944478113	-2.685330124
C	-12.10926728	-5.541858676	-1.220226869
H	-11.40439557	-6.117722242	-0.580761019
H	-12.81646874	-4.9922079	-0.562990734
C	-12.8911528	-6.507276235	-2.120636338
H	-13.64904015	-5.93550896	-2.699843332
H	-12.19172962	-6.990643925	-2.838243802
C	-13.58883417	-7.595272236	-1.300294913

H	-14.30331795	-7.122040618	-0.590815879
H	-12.82498303	-8.159621712	-0.72485979
C	-14.33549167	-8.575629984	-2.209291627
H	-15.05997706	-8.023165283	-2.842286039
H	-13.61250046	-9.090081245	-2.879125005
C	-15.08285702	-9.62444651	-1.391626141
H	-15.79955505	-9.136688142	-0.698347865
H	-15.6482708	-10.29406169	-2.073145593
H	-14.36568922	-10.23644447	-0.804569777
C	-3.606677369	14.36944906	2.432069362
H	-4.453986334	14.69126101	3.077129095
H	-2.785028062	14.00118813	3.085446346
C	-3.106847244	15.56765749	1.616442208
H	-3.928508471	15.9367121	0.963565366
H	-2.259898697	15.24558991	0.971035927
C	-2.640589785	16.70296772	2.535538467
H	-3.487878028	17.02576496	3.180149251
H	-1.819874617	16.33335311	3.189331595
C	-2.1387063	17.90079612	1.720667147
H	-2.95924294	18.27097439	1.066959508
H	-1.291183779	17.57828705	1.076188799
C	-1.672711034	19.03508758	2.640560659
H	-2.516167669	19.36561329	3.285307
H	-0.849942996	18.67317492	3.295117002
C	-1.171699889	20.22912436	1.833335781
H	-1.983779032	20.62675391	1.187971143
H	-0.841825549	21.03303704	2.524629299
H	-0.310354303	19.93141679	1.198089256
C	16.9379448	-2.529799686	7.642607711
H	17.83576385	-2.125670431	7.124681802
H	17.09431061	-3.615916055	7.825627487
C	16.76002262	-1.809946015	8.984574403
H	16.60335377	-0.723896401	8.801394135
H	15.86235783	-2.214237102	9.502598453
C	17.99052507	-2.000798884	9.879190145
H	18.14719953	-3.086857261	10.06235343
H	18.88817128	-1.596524936	9.361103016
C	17.81276276	-1.28096861	11.22119178

H	17.65619002	-0.194828567	11.03831129
H	16.91533243	-1.685303155	11.73964732
C	19.04340139	-1.472095301	12.11499631
H	19.20409862	-2.555758026	12.3052947
H	19.94426312	-1.066763696	11.60458948
C	18.87032584	-0.756977979	13.45179125
H	17.98979742	-1.161764447	13.99484241
H	19.77502935	-0.912674334	14.07648418
H	18.73307198	0.333632029	13.29111283
C	21.27935103	3.95248881	-3.133941505
H	21.13936543	4.710971326	-2.334664163
H	22.3160096	3.554400945	-3.069550158
C	21.06618363	4.605187963	-4.508030233
H	21.12566663	3.818392298	-5.291905776
H	20.05054094	5.055439463	-4.560235168
C	22.12596916	5.671150295	-4.828347241
H	23.14356403	5.247753489	-4.679157118
H	22.02746583	5.938508478	-5.903458595
C	21.95969429	6.943859513	-3.984657337
H	22.1422456	6.710039499	-2.914157329
H	20.92125401	7.327373329	-4.093293246
C	22.94778979	8.02865685	-4.428945513
H	23.9889176	7.652023911	-4.325883866
H	22.76990194	8.284514195	-5.49629079
C	22.79454652	9.292606439	-3.587756414
H	22.99400611	9.070707807	-2.517681311
H	23.52042462	10.05886479	-3.932595131
H	21.76775406	9.703526009	-3.692281489
C	11.09240716	-3.718344222	2.026784265
H	11.14274029	-3.545818947	0.9323681
H	10.35027763	-4.52997386	2.193182676
C	10.59459584	-2.445319166	2.731774083
H	10.67836869	-2.569997584	3.833734052
H	11.21823718	-1.578480461	2.428557403
C	9.13099141	-2.156535246	2.374102171
H	9.023249859	-2.094397805	1.269352755
H	8.495394321	-2.991990762	2.742111998
C	8.656380585	-0.841263081	3.00332266

H	8.834688061	-0.870224395	4.100909862
H	9.241589274	0.001181956	2.573998919
C	7.162310652	-0.613043424	2.744642193
H	6.967270756	-0.629390176	1.65130628
H	6.579106447	-1.434878063	3.215575795
C	6.703508956	0.73039666	3.322178379
H	6.905191905	0.758182945	4.41510861
H	7.267357269	1.557814652	2.839112976
C	5.211780374	0.94830705	3.091165637
H	4.620769817	0.154438901	3.595492237
H	4.909601247	1.93470023	3.501296587
H	4.989030418	0.933580528	2.006364509
H	12.35128876	-4.305497484	3.67404026
C	19.29959856	-0.050140884	-2.205205415
H	18.48713543	-0.736413051	-1.89350804
H	18.96597574	0.357404797	-3.182994961
C	20.60139638	-0.842672923	-2.41846547
H	21.35078727	-0.202215998	-2.93051041
H	21.0230225	-1.157042558	-1.443617002
C	20.35098444	-2.088474673	-3.275734521
H	19.54877993	-2.701599409	-2.809938311
H	20.01064368	-1.77753957	-4.28793921
C	21.62428737	-2.933344629	-3.401043212
H	21.99812235	-3.191545516	-2.385685265
H	22.40747549	-2.341921536	-3.924556042
C	21.35302784	-4.22553175	-4.179164335
H	20.55809385	-4.806469748	-3.660969105
H	20.9965415	-3.971198247	-5.201732906
C	22.61997559	-5.082805805	-4.279137429
H	22.97639803	-5.332475145	-3.255150799
H	23.41527489	-4.504586751	-4.799422298
C	22.3477099	-6.379478929	-5.050506038
H	21.55086343	-6.956154441	-4.530748547
H	21.99289818	-6.130366438	-6.075026273
C	23.61374754	-7.238779832	-5.147144404
H	23.96874649	-7.487533125	-4.122553449
H	24.41067725	-6.662733406	-5.667456529
C	23.34064068	-8.53563048	-5.917485909

H	22.54702348	-9.11860659	-5.401075202
H	22.9889673	-8.294870104	-6.944480084
C	24.59839954	-9.394004974	-6.015393244
H	24.37067958	-10.32434397	-6.577160044
H	25.40083066	-8.843166099	-6.550897085
H	24.95688942	-9.670445586	-5.000919864
H	-7.000232344	9.026054614	-1.368491844
C	-8.999701149	8.182890753	-1.151798846
H	-9.005808849	7.715310848	-2.16089821
H	-9.541953385	7.499144253	-0.461973748
C	-9.722282423	9.535474101	-1.224115745
H	-9.791359609	9.975809621	-0.206139417
H	-9.144365253	10.22914986	-1.873737021
C	-11.13750372	9.370639685	-1.790554581
H	-11.07655294	8.938875437	-2.814006784
H	-11.71230866	8.67015285	-1.14504898
C	-11.8667823	10.71784804	-1.852130452
H	-11.29160786	11.41847296	-2.497075604
H	-11.92937147	11.14966086	-0.828761024
C	-13.28118319	10.5518779	-2.419350672
H	-13.86330164	9.855025631	-1.777546456
H	-13.22648238	10.12417775	-3.444262139
C	-14.01065556	11.89063736	-2.481908524
H	-15.02986968	11.73958329	-2.895855119
H	-14.10162479	12.32843858	-1.464981779
H	-13.46190412	12.59880496	-3.13876537
H	-5.980210713	-0.084736039	1.224145495
C	-3.948141943	0.453843984	0.613369518
H	-3.70670986	1.024389367	1.53613543
H	-3.463402797	0.994062643	-0.229142277
C	-3.341576305	-0.956514424	0.715213365
H	-3.640083672	-1.573559151	-0.158656282
H	-2.23581708	-0.850922204	0.678227239
C	-3.729758974	-1.66762305	2.019978096
H	-3.508188632	-1.003533187	2.883932194
H	-4.817969263	-1.890610081	2.015453988
C	-2.959527721	-2.982136361	2.18510315
H	-3.215549286	-3.66397198	1.344380803

H	-1.86676541	-2.776975328	2.157136718
C	-3.311877726	-3.656678054	3.516102146
H	-4.403150979	-3.866770009	3.553230013
H	-3.055407871	-2.977691676	4.358480558
C	-2.551176708	-4.966855422	3.69339958
H	-2.808269615	-5.415566001	4.675907245
H	-2.830431473	-5.684468154	2.893904558
H	-1.455522799	-4.785746278	3.661508081

11. References

S1. C.-H. Lee, Y.-Y. Lai, S.-W. Cheng and Y.-J. Cheng, *Org. Lett.*, 2014, **16**, 936-939.

S2. A. T. Yiu, P. M. Beaujuge, O. P. Lee, C. H. Woo, M. F. Toney and J. M. J. Fréchet, *J. Am. Chem. Soc.*, 2012, **134**, 2180-2185.

S3. M. L. Chabinyc, M. F. Toney, R. J. Kline, I. McCulloch and M. Heeney, *J. Am. Chem. Soc.*, 2007, **129**, 3226-3237.

S4. N. Zhou, A. S. Dudnik, T. I. N. G. Li, E. F. Manley, T. J. Aldrich, P. Guo, H.-C. Liao, Z. Chen, L. X. Chen, R. P. H. Chang, A. Facchetti, M. Olvera de la Cruz and T. J. Marks, *J. Am. Chem. Soc.*, 2016, **138**, 1240-1251.

S5. M. S. Chen, O. P. Lee, J. R. Niskala, A. T. Yiu, C. J. Tassone, K. Schmidt, P. M. Beaujuge, S. S. Onishi, M. F. Toney, A. Zettl and J. M. J. Fréchet, *J. Am. Chem. Soc.*, 2013, **135**, 19229-19236.

S6. Gaussian 09, Revision D.01, Frisch, M. J.; Trucks, G. W.; Schlegel, H. B.; Scuseria, G. E.; Robb, M. A.; Cheeseman, J. R.; Scalmani, G.; Barone, V.; Mennucci, B.; Petersson, G. A.; Nakatsuji, H.; Caricato, M.; Li, X.; Hratchian, H. P.; Izmaylov, A. F.; Bloino, J.; Zheng, G.; Sonnenberg, J. L.; Hada, M.; Ehara, M.; Toyota, K.; Fukuda, R.; Hasegawa, J.; Ishida, M.; Nakajima, T.; Honda, Y.; Kitao, O.; Nakai, H.; Vreven, T.; Montgomery, J. A., Jr.; Peralta, J. E.; Ogliaro, F.; Bearpark, M.; Heyd, J. J.; Brothers, E.; Kudin, K. N.; Staroverov, V. N.; Kobayashi, R.; Normand, J.; Raghavachari, K.; Rendell, A.; Burant, J. C.; Iyengar, S. S.; Tomasi, J.; Cossi, M.; Rega, N.; Millam, J. M.; Klene, M.; Knox, J. E.; Cross, J. B.; Bakken, V.; Adamo, C.; Jaramillo, J.; Gomperts, R.; Stratmann, R. E.; Yazyev, O.; Austin, A. J.; Cammi, R.; Pomelli, C.; Ochterski, J. W.; Martin, R. L.; Morokuma, K.; Zakrzewski, V. G.; Voth, G. A.; Salvador, P.; Dannenberg, J. J.; Dapprich, S.; Daniels, A. D.; Farkas, Ö.; Foresman, J. B.; Ortiz, J. V.; Cioslowski, J.; Fox, D. J. Gaussian, Inc., Wallingford CT, 2009.
